# Supplementary material for: Accuracy of immunological tests on serum and urine for diagnosis of Taenia solium neurocysticercosis: A systematic review
Source: PLoS Negl Trop Dis. 2024 Nov 11;18(11):e0012643. doi: 10.1371/journal.pntd.0012643 (PMC11581404; doi:10.1371/journal.pntd.0012643)
Supplement: S1 Search Strategy — (DOCX) [file pntd.0012643.s002.docx]

**S1 Search strategy. Search strategy for databases searched.**

**EMBASE**

| **#** | **Search terms** |
| --- | --- |
| 1 | 'neurocysticercosis'/exp OR neurocysticerc*:ti,ab OR neurocisticerc*:ti,ab |
| 2 | 'human cysticerc*':ti,ab OR 'human cisticerc*':ti,ab |
| 3 | 'cysticercosis'/exp OR cysticerc*:ti,ab OR cisticerc*:ti,ab |
| 4 | cyst:ti,ab OR 'cystic lesion':ti,ab OR 'cystical lesion':ti,ab OR cestod*:ti,ab OR metacestod*:ti,ab |
| 5 | 'taenia solium'/exp OR 'taenia solium':ti,ab OR 'tenia solium':ti,ab OR 't. solium':ti,ab |
| 6 | #4 AND #5 |
| 7 | #3 OR #6 |
| 8 | brain:ti,ab OR cerebral:ti,ab OR 'central nervous':ti,ab OR 'central nervous system':ti,ab OR 'cns':ti,ab OR neuro*:ti,ab OR neural:ti,ab OR medullary:ti,ab OR intramedullary:ti,ab OR 'intra medullary':ti,ab OR extramedullary:ti,ab OR 'extra medullary':ti,ab OR ventricular:ti,ab OR subarachnoid*:ti,ab OR parenchymal:ti,ab OR intraparenchymal:ti,ab OR 'intra parenchymal':ti,ab OR extraparenchymal:ti,ab OR 'extra parenchymal':ti,ab OR spinal:ti,ab OR spine:ti,ab OR racemose:ti,ab |
| 9 | #7 AND #8 |
| 10 | #1 OR #2 OR #9 |
| 11 | 'immunoblotting'/exp OR 'enzyme linked immunoelectrotransfer':ti,ab OR 'enzyme linked immunoelectro transfer':ti,ab OR 'enzyme linked immuno electrotransfer':ti,ab OR 'enzyme linked immuno-electrotransfer':ti,ab OR 'enzyme linked immuno-electro transfer':ti,ab OR 'enzyme linked immunoelectro-transfer':ti,ab OR 'enzyme linked immuno electro-transfer':ti,ab OR 'enzyme linked immuno-electro-transfer':ti,ab OR 'enzyme linked immune electrotransfer':ti,ab OR 'enzyme linked immune-electrotransfer':ti,ab OR 'enzyme linked immune-electro transfer':ti,ab OR 'enzyme linked immune electro-transfer':ti,ab OR 'enzyme linked immune-electro-transfer':ti,ab OR 'enzyme-linked immunoelectrotransfer':ti,ab OR 'enzyme-linked immunoelectro transfer':ti,ab OR 'enzyme-linked immuno electrotransfer':ti,ab OR 'enzyme-linked immuno-electrotransfer':ti,ab OR 'enzyme-linked immuno-electro transfer':ti,ab OR 'enzyme-linked immunoelectro-transfer':ti,ab OR 'enzyme-linked immuno electro-transfer':ti,ab OR 'enzyme-linked immuno-electro-transfer':ti,ab OR 'enzyme-linked immune electrotransfer':ti,ab OR 'enzyme-linked immune-electrotransfer':ti,ab OR 'enzyme-linked immune-electro transfer':ti,ab OR 'enzyme-linked immune electro-transfer':ti,ab OR 'enzyme-linked immune-electro-transfer':ti,ab OR 'enzyme linked-immunoelectrotransfer':ti,ab OR 'enzyme linked-immunoelectro transfer':ti,ab OR 'enzyme linked-immuno electrotransfer':ti,ab OR 'enzyme linked-immuno-electrotransfer':ti,ab OR 'enzyme linked-immuno-electro transfer':ti,ab OR 'enzyme linked-immunoelectro-transfer':ti,ab OR 'enzyme linked-immuno electro-transfer':ti,ab OR 'enzyme linked-immuno-electro-transfer':ti,ab OR 'enzyme linked-immune electrotransfer':ti,ab OR 'enzyme linked-immune-electrotransfer':ti,ab OR 'enzyme linked-immune-electro transfer':ti,ab OR 'enzyme linked-immune electro-transfer':ti,ab OR 'enzyme linked-immune-electro-transfer':ti,ab OR 'enzyme-linked-immunoelectrotransfer':ti,ab OR 'enzyme-linked-immunoelectro transfer':ti,ab OR 'enzyme-linked-immuno electrotransfer':ti,ab OR 'enzyme-linked-immuno-electrotransfer':ti,ab OR 'enzyme-linked-immuno-electro transfer':ti,ab OR 'enzyme-linked-immunoelectro-transfer':ti,ab OR 'enzyme-linked-immuno electro-transfer':ti,ab OR 'enzyme-linked-immuno-electro-transfer':ti,ab OR 'enzyme-linked-immune electrotransfer':ti,ab OR 'enzyme-linked-immune-electrotransfer':ti,ab OR 'enzyme-linked-immune-electro transfer':ti,ab OR 'enzyme-linked-immune electro-transfer':ti,ab OR 'enzyme-linked-immune-electro-transfer':ti,ab OR immunoblot:ti,ab OR immunoblotting:ti,ab OR 'immuno blot':ti,ab OR 'immuno blotting':ti,ab OR immuneblot:ti,ab OR immuneblotting:ti,ab OR 'immune blot':ti,ab OR 'immune blotting':ti,ab OR electroimmunotransfer:ti,ab OR 'electro immunotransfer':ti,ab OR 'electroimmuno transfer':ti,ab OR 'electro immuno transfer':ti,ab OR 'electro-immunotransfer':ti,ab OR 'electro-immuno transfer':ti,ab OR 'electro immuno-transfer':ti,ab OR 'electro-immuno-transfer':ti,ab OR electroimmunetransfer:ti,ab OR 'electro immunetransfer':ti,ab OR 'electroimmune transfer':ti,ab OR 'electro immune transfer':ti,ab OR 'electro-immunetransfer':ti,ab OR 'electro-immune transfer':ti,ab OR 'electro immune-transfer':ti,ab OR 'electro-immune-transfer':ti,ab OR 'western blot':ti,ab OR 'western blotting':ti,ab OR 'DOT blot':ti,ab OR 'DOT blotting':ti,ab OR 'EITB':ti,ab |

| 12 | 'enzyme linked immunosorbent assay'/exp OR 'elisa':ti,ab OR 'eia':ti,ab OR 'enzyme linked immunosorbent':ti,ab OR 'enzyme linked immuno sorbent':ti,ab OR 'enzyme linked immuno-sorbent':ti,ab OR 'enzyme linked immunesorbent':ti,ab OR 'enzyme linked immune sorbent':ti,ab OR 'enzyme linked immune-sorbent':ti,ab OR 'enzyme-linked immunosorbent':ti,ab OR 'enzyme-linked immuno sorbent':ti,ab OR 'enzyme-linked immuno-sorbent':ti,ab OR 'enzyme-linked immunesorbent':ti,ab OR 'enzyme-linked immune sorbent':ti,ab OR 'enzyme-linked immune-sorbent':ti,ab OR 'enzyme linked-immunosorbent':ti,ab OR 'enzyme linked-immuno sorbent':ti,ab OR 'enzyme linked-immuno-sorbent':ti,ab OR 'enzyme linked-immunesorbent':ti,ab OR 'enzyme linked-immune sorbent':ti,ab OR 'enzyme linked-immune-sorbent':ti,ab OR 'enzyme-linked-immunosorbent':ti,ab OR 'enzyme-linked-immuno sorbent':ti,ab OR 'enzyme-linked-immuno-sorbent':ti,ab OR 'enzyme-linked-immunesorbent':ti,ab OR 'enzyme-linked-immune sorbent':ti,ab OR 'enzyme-linked-immune-sorbent':ti,ab OR 'enzyme-immuno assay':ti,ab OR 'enzyme-immunoassay':ti,ab OR 'enzyme-immuno-assay':ti,ab OR 'enzyme-immune assay':ti,ab OR 'enzyme-immune-assay':ti,ab OR 'dot elisa':ti,ab OR 'dot-elisa':ti,ab OR 'ag elisa':ti,ab OR 'ag-elisa':ti,ab OR 'ab elisa':ti,ab OR 'ab-elisa':ti,ab |
| --- | --- |
| 13 | 'multiplex':ti,ab OR 'multiple bead':ti,ab OR 'multiple-based':ti,ab OR 'bead based':ti,ab OR 'bead-based':ti,ab |
| 14 | 'point of care testing'/exp OR 'point-of-care test*':ti,ab OR 'point of care test*':ti,ab OR 'poc':ti,ab |
| 15 | 'immunological procedures'/exp OR immunoassay:ti,ab OR 'immuno assay':ti,ab OR 'immuno-assay':ti,ab OR 'immune assay':ti,ab OR 'immune-assay':ti,ab OR immunologic*:ti,ab OR immunodiagnosis:ti,ab OR immunodiagnostic*:ti,ab OR immunodetect*:ti,ab OR 'serology'/exp OR serodiagnosis:ti,ab OR serodiagnostic*:ti,ab OR 'antigen assay':ti,ab OR 'antigen-assay':ti,ab OR 'antibody assay':ti,ab OR 'antibody-assay':ti,ab |
| 16 | 'diagnostic marker':ti,ab OR 'diagnostic antigen':ti,ab OR 'diagnostic antibody':ti,ab OR 'diagnostic protein':ti,ab OR 'diagnostic peptide':ti,ab OR 'diagnostic reagent':ti,ab OR 'immun* marker':ti,ab OR 'immun* antigen':ti,ab OR 'immun* antibody':ti,ab OR 'immun* protein':ti,ab OR 'immun* peptide':ti,ab OR 'immun* reagent':ti,ab OR biomarker:ti,ab OR biosensor:ti,ab OR mimotope:ti,ab OR 'antigen diagnos*':ti,ab OR 'antibody diagnos*':ti,ab |
| 17 | #11 or #12 or #13 or #14 or #15 or #16 |
| 18 | 'PCR':ti,ab OR 'qPCR':ti,ab OR 'polymerase chain reaction':ti,ab OR 'polymerase-chain reaction':ti,ab OR 'polymerase chain-reaction':ti,ab OR 'polymerase-chain-reaction':ti,ab |
| 19 | #17 NOT #18 |
| 20 | sensitiv*:ti,ab OR specific*:ti,ab OR predict*:ti,ab OR evaluat*:ti,ab OR accurate:ti,ab OR accuracy:ti,ab OR valid*:ti,ab OR perform*:ti,ab OR 'cross react*':ti,ab OR 'cross-react*':ti,ab OR utili*:ti,ab OR applic*:ti,ab |
| 21 | urine:ti,ab OR urin*:ti,ab OR serolog*:ti,ab OR serum:ti,ab OR sera:ti,ab OR blood:ti,ab OR plasma:ti,ab OR antigen*:ti,ab OR antibody:ti,ab OR antibodies:ti,ab |
| 22 | #10 AND #19 AND #20 AND #21 |

**Scopus**

| **#** | **Search terms** |
| --- | --- |
| 1 | INDEXTERMS ( 'neurocysticercosis' ) OR TITLE-ABS ( neurocysticerc* ) OR TITLE-ABS ( neurocisticerc* ) |
| 2 | TITLE-ABS ( "human cysticerc*" ) OR TITLE-ABS ( "human cisticerc*" ) |
| 3 | INDEXTERMS ( 'cysticercosis' )  OR  TITLE-ABS ( cysticerc* )  OR  TITLE-ABS ( cisticerc* ) |
| 4 | TITLE-ABS ( cyst ) OR TITLE-ABS ( "cystic lesion" ) OR TITLE-ABS ( "cystical lesion" ) OR TITLE-ABS ( cestod* ) OR TITLE-ABS ( metacestod* ) |
| 5 | TITLE-ABS ( "taenia solium" ) OR TITLE-ABS ( "tenia solium" ) OR TITLE-ABS ( "T. solium" ) OR INDEXTERMS ( "taenia solium" ) |
| 6 | #4 AND #5 |
| 7 | #3 OR #6 |
| 8 | TITLE-ABS ( brain ) OR TITLE-ABS ( cerebral ) OR TITLE-ABS ( "central nervous" ) OR TITLE-ABS ( "central nervous system" ) OR TITLE-ABS ( "CNS" ) OR TITLE-ABS ( neuro* ) OR TITLE-ABS ( neural ) OR TITLE-ABS ( medullary ) OR TITLE-ABS ( intramedullary ) OR TITLE-ABS ( intra-medullary ) OR TITLE-ABS ( extramedullary ) OR TITLE-ABS ( extra-medullary ) OR TITLE-ABS ( ventricular ) OR TITLE-ABS ( subarachnoid* ) OR TITLE-ABS ( parenchymal ) OR TITLE-ABS ( intraparenchymal ) OR TITLE-ABS ( intra-parenchymal ) OR TITLE-ABS ( extraparenchymal ) OR TITLE-ABS ( extra-parenchymal ) OR TITLE-ABS ( spinal ) OR TITLE-ABS ( spine ) OR TITLE-ABS ( racemose ) |
| 9 | #7 AND #8 |
| 10 | #1 OR #2 OR #9 |
| 11 | INDEXTERMS ( "Immunoblotting" ) OR TITLE-ABS ( "enzyme linked immunoelectrotransfer" ) OR TITLE-ABS ( "enzyme linked immunoelectro transfer" ) OR TITLE-ABS ( "enzyme linked immuno electrotransfer" ) OR TITLE-ABS ( "enzyme linked immuno-electrotransfer" ) OR TITLE-ABS ( "enzyme linked immuno-electro transfer" ) OR TITLE-ABS ( "enzyme linked immunoelectro-transfer" ) OR TITLE-ABS ( "enzyme linked immuno electro-transfer" ) OR TITLE-ABS ( "enzyme linked immuno-electro-transfer" ) OR TITLE-ABS ( "enzyme linked immune electrotransfer" ) OR TITLE-ABS ( "enzyme linked immune-electrotransfer" ) OR TITLE-ABS ( "enzyme linked immune-electro transfer" ) OR TITLE-ABS ( "enzyme linked immune electro-transfer" ) OR TITLE-ABS ( "enzyme linked immune-electro-transfer" ) OR TITLE-ABS ( "enzyme-linked immunoelectrotransfer" ) OR TITLE-ABS ( "enzyme-linked immunoelectro transfer" ) OR TITLE-ABS ( "enzyme-linked immuno electrotransfer" ) OR TITLE-ABS ( "enzyme-linked immuno-electrotransfer" ) OR TITLE-ABS ( "enzyme-linked immuno-electro transfer" ) OR TITLE-ABS ( "enzyme-linked immunoelectro-transfer" ) OR TITLE-ABS ( "enzyme-linked immuno electro-transfer" ) OR TITLE-ABS ( "enzyme-linked immuno-electro-transfer" ) OR TITLE-ABS ( "enzyme-linked immune electrotransfer" ) OR TITLE-ABS ( "enzyme-linked immune-electrotransfer" ) OR TITLE-ABS ( "enzyme-linked immune-electro transfer" ) OR TITLE-ABS ( "enzyme-linked immune electro-transfer" ) OR TITLE-ABS ( "enzyme-linked immune-electro-transfer" ) OR TITLE-ABS ( "enzyme linked-immunoelectrotransfer" ) OR TITLE-ABS ( "enzyme linked-immunoelectro transfer" ) OR TITLE-ABS ( "enzyme linked-immuno electrotransfer" ) OR TITLE-ABS ( "enzyme linked-immuno-electrotransfer" ) OR TITLE-ABS ( "enzyme linked-immuno-electro transfer" ) OR TITLE-ABS ( "enzyme linked-immunoelectro-transfer" ) OR TITLE-ABS ( "enzyme linked-immuno electro-transfer" ) OR TITLE-ABS ( "enzyme linked-immuno-electro-transfer" ) OR TITLE-ABS ( "enzyme linked-immune electrotransfer" ) OR TITLE-ABS ( "enzyme linked-immune-electrotransfer" ) OR TITLE-ABS ( "enzyme linked-immune-electro transfer" ) OR TITLE-ABS ( "enzyme linked-immune electro-transfer" ) OR TITLE-ABS ( "enzyme linked-immune-electro-transfer" ) OR TITLE-ABS ( "enzyme-linked-immunoelectrotransfer" ) OR TITLE-ABS ( "enzyme-linked-immunoelectro transfer" ) OR TITLE-ABS ( "enzyme-linked-immuno electrotransfer" ) OR TITLE-ABS ( "enzyme-linked-immuno-electrotransfer" ) OR TITLE-ABS ( "enzyme-linked-immuno-electro transfer" ) OR TITLE-ABS ( "enzyme-linked-immunoelectro-transfer" ) OR TITLE-ABS ( "enzyme-linked-immuno electro-transfer" ) OR TITLE-ABS ( "enzyme-linked-immuno-electro-transfer" ) OR TITLE-ABS ( "enzyme-linked-immune electrotransfer" ) OR TITLE-ABS ( "enzyme-linked-immune-electrotransfer" ) OR TITLE-ABS ( "enzyme-linked-immune-electro transfer" ) OR TITLE-ABS ( "enzyme-linked-immune electro-transfer" ) OR TITLE-ABS ( "enzyme-linked-immune-electro-transfer" ) OR TITLE-ABS ( immunoblot ) OR TITLE-ABS ( immunoblotting ) OR TITLE-ABS ( "immuno blot" ) OR TITLE-ABS ( "immuno blotting" ) OR TITLE-ABS ( immuneblot ) OR TITLE-ABS ( immuneblotting ) OR TITLE-ABS ( "immune blot" ) OR TITLE-ABS ( "immune blotting" ) OR TITLE-ABS ( electroimmunotransfer ) OR TITLE-ABS ( "electro immunotransfer" ) OR TITLE-ABS ( "electroimmuno transfer" ) OR TITLE-ABS ( "electro immuno transfer" ) OR TITLE-ABS ( "electro-immunotransfer" ) OR TITLE-ABS ( "electro-immuno transfer" ) OR TITLE-ABS ( "electro immuno-transfer" ) OR TITLE-ABS ( "electro-immuno-transfer" ) OR TITLE-ABS ( "electroimmunetransfer" ) OR TITLE-ABS ( "electro immunetransfer" ) OR TITLE-ABS ( "electroimmune transfer" ) OR TITLE-ABS ( "electro immune transfer" ) OR TITLE-ABS ( "electro-immunetransfer" ) OR TITLE-ABS ( "electro-immune transfer" ) OR TITLE-ABS ( "electro immune-transfer" ) OR TITLE-ABS ( "electro-immune-transfer" ) OR TITLE-ABS ( "western blot" ) OR TITLE-ABS ( "western blotting" ) OR TITLE-ABS ( "DOT blot" ) OR TITLE-ABS ( "DOT blotting" ) OR TITLE-ABS ( "EITB" ) |

| 12 | INDEXTERMS("Enzyme-Linked Immunosorbent Assay") OR TITLE-ABS("ELISA") OR TITLE-ABS("EIA") OR TITLE-ABS("enzyme linked immunosorbent") OR TITLE-ABS("enzyme linked immuno sorbent") OR TITLE-ABS("enzyme linked immuno-sorbent") OR TITLE-ABS("enzyme linked immunesorbent") OR TITLE-ABS("enzyme linked immune sorbent") OR TITLE-ABS("enzyme linked immune-sorbent") OR TITLE-ABS("enzyme-linked immunosorbent") OR TITLE-ABS("enzyme-linked immuno sorbent") OR TITLE-ABS("enzyme-linked immuno-sorbent") OR TITLE-ABS("enzyme-linked immunesorbent") OR TITLE-ABS("enzyme-linked immune sorbent") OR TITLE-ABS("enzyme-linked immune-sorbent") OR TITLE-ABS("enzyme linked-immunosorbent") OR TITLE-ABS("enzyme linked-immuno sorbent") OR TITLE-ABS("enzyme linked-immuno-sorbent") OR TITLE-ABS("enzyme linked-immunesorbent") OR TITLE-ABS("enzyme linked-immune sorbent") OR TITLE-ABS("enzyme linked-immune-sorbent") OR TITLE-ABS("enzyme-linked-immunosorbent") OR TITLE-ABS("enzyme-linked-immuno sorbent") OR TITLE-ABS("enzyme-linked-immuno-sorbent") OR TITLE-ABS("enzyme-linked-immunesorbent") OR TITLE-ABS("enzyme-linked-immune sorbent") OR TITLE-ABS("enzyme-linked-immune-sorbent") OR TITLE-ABS("enzyme-immuno assay") OR TITLE-ABS("enzyme-immunoassay") OR TITLE-ABS("enzyme-immuno-assay") OR TITLE-ABS("enzyme-immune assay") OR TITLE-ABS("enzyme-immune-assay") OR TITLE-ABS("DOT ELISA") OR TITLE-ABS("DOT-ELISA") OR TITLE-ABS("Ag ELISA") OR TITLE-ABS("Ag-ELISA") OR TITLE-ABS("Ab ELISA") OR TITLE-ABS("Ab-ELISA") |
| --- | --- |
| 13 | TITLE-ABS(multiplex) OR TITLE-ABS("multiple bead") OR TITLE-ABS("multiple-bead") OR TITLE-ABS("bead based") OR TITLE-ABS("bead-based") |
| 14 | INDEXTERMS("Point-of-Care Testing") OR TITLE-ABS("point-of-care test*") OR TITLE-ABS("point of care test*") OR TITLE-ABS("POC") |
| 15 | INDEXTERMS("Immunologic* Test*") OR TITLE-ABS(immunoassay) OR TITLE-ABS("immuno assay") OR TITLE-ABS("immuno-assay") OR TITLE-ABS("immune assay") OR TITLE-ABS("immune-assay") OR TITLE-ABS(immunologic*) OR TITLE-ABS(immunodiagnosis) OR TITLE-ABS(immunodiagnostic*) OR TITLE-ABS(immunodetect*) OR INDEXTERMS("Serologic* Test*") OR TITLE-ABS(serodiagnosis) OR TITLE-ABS(serodiagnostic*) OR TITLE-ABS("antigen assay") OR TITLE-ABS("antigen-assay") OR TITLE-ABS("antibody assay") OR TITLE-ABS("antibody-assay") |
| 16 | TITLE-ABS("diagnostic marker") OR TITLE-ABS("diagnostic antigen") OR TITLE-ABS("diagnostic antibody") OR TITLE-ABS("diagnostic protein") OR TITLE-ABS("diagnostic peptide") OR TITLE-ABS("diagnostic reagent") OR TITLE-ABS("immun* marker") OR TITLE-ABS("immun* antigen") OR TITLE-ABS("immun* antibody") OR TITLE-ABS("immun* protein") OR TITLE-ABS("immun* peptide") OR TITLE-ABS("immun* reagent") OR TITLE-ABS(biomarker) OR TITLE-ABS(biosensor) OR TITLE-ABS(mimotope) OR TITLE-ABS("antigen diagnos*") OR TITLE-ABS("antibody diagnos*") |
| 17 | #11 OR #12 OR #13 OR #14 OR #15 OR #16 |
| 18 | TITLE-ABS(PCR) OR TITLE-ABS(qPCR) OR TITLE-ABS("polymerase chain reaction") OR TITLE-ABS("polymerase-chain reaction") OR TITLE-ABS("polymerase chain-reaction") OR TITLE-ABS("polymerase-chain-reaction") |
| 19 | #17 NOT #18 |
| 20 | TITLE-ABS(sensitiv*) OR TITLE-ABS(specific*) OR TITLE-ABS(predict*) OR TITLE-ABS(evaluat*) OR TITLE-ABS(accurate) OR TITLE-ABS(accuracy) OR TITLE-ABS(valid*) OR TITLE-ABS(perform*) OR TITLE-ABS("cross react*") OR TITLE-ABS("cross-react*") OR TITLE-ABS(utili*) OR TITLE-ABS(applic*) |
| 21 | TITLE-ABS(urine) OR TITLE-ABS(urin*) OR TITLE-ABS(serolog*) OR TITLE-ABS(serum) OR TITLE-ABS(sera) OR TITLE-ABS(blood) OR TITLE-ABS(plasma) OR TITLE-ABS(antigen*) OR TITLE-ABS(antibody) OR TITLE-ABS(antibodies) |
| 22 | #10 AND #19 AND #20 AND #21 |

**Web of Science**

| **#** | **Search terms** |
| --- | --- |
| 1 | TS=(neurocysticerc*) OR TS=(neurocisticerc*) |
| 2 | TS=("human cysticerc*") OR TS=("human cisticerc*") |
| 3 | TS=(cysticerc*)  OR  TS=(cisticerc*) |
| 4 | TS=( cyst ) OR TS=( "cystic lesion" ) OR TS=( "cystical lesion" ) OR TS=( cestod* ) OR TS=( metacestod* ) |
| 5 | TS=( "taenia solium" ) OR TS=( "tenia solium" ) OR TS=( "T. solium" ) |
| 6 | #4 AND #5 |
| 7 | #3 OR #6 |
| 8 | TS=( brain ) OR TS=( cerebral ) OR TS=( "central nervous" ) OR TS=( "central nervous system" ) OR TS=( "CNS" ) OR TS=( neuro* ) OR TS=( neural ) OR TS=( medullary ) OR TS=( intramedullary ) OR TS=( intra-medullary ) OR TS=( extramedullary ) OR TS=( extra-medullary ) OR TS=( ventricular ) OR TS=( subarachnoid* ) OR TS=( parenchymal ) OR TS=( intraparenchymal ) OR TS=( intra-parenchymal ) OR TS=( extraparenchymal ) OR TS=( extra-parenchymal ) OR TS=( spinal ) OR TS=( spine ) OR TS=( racemose ) |
| 9 | #7 AND #8 |
| 10 | #1 OR #2 OR #9 |
| 11 | TS=( "enzyme linked immunoelectrotransfer" ) OR TS=( "enzyme linked immunoelectro transfer" ) OR TS=( "enzyme linked immuno electrotransfer" ) OR TS=( "enzyme linked immuno-electrotransfer" ) OR TS=( "enzyme linked immuno-electro transfer" ) OR TS=( "enzyme linked immunoelectro-transfer" ) OR TS=( "enzyme linked immuno electro-transfer" ) OR TS=( "enzyme linked immuno-electro-transfer" ) OR TS=( "enzyme linked immune electrotransfer" ) OR TS=( "enzyme linked immune-electrotransfer" ) OR TS=( "enzyme linked immune-electro transfer" ) OR TS=( "enzyme linked immune electro-transfer" ) OR TS=( "enzyme linked immune-electro-transfer" ) OR TS=( "enzyme-linked immunoelectrotransfer" ) OR TS=( "enzyme-linked immunoelectro transfer" ) OR TS=( "enzyme-linked immuno electrotransfer" ) OR TS=( "enzyme-linked immuno-electrotransfer" ) OR TS=( "enzyme-linked immuno-electro transfer" ) OR TS=( "enzyme-linked immunoelectro-transfer" ) OR TS=( "enzyme-linked immuno electro-transfer" ) OR TS=( "enzyme-linked immuno-electro-transfer" ) OR TS=( "enzyme-linked immune electrotransfer" ) OR TS=( "enzyme-linked immune-electrotransfer" ) OR TS=( "enzyme-linked immune-electro transfer" ) OR TS=( "enzyme-linked immune electro-transfer" ) OR TS=( "enzyme-linked immune-electro-transfer" ) OR TS=( "enzyme linked-immunoelectrotransfer" ) OR TS=( "enzyme linked-immunoelectro transfer" ) OR TS=( "enzyme linked-immuno electrotransfer" ) OR TS=( "enzyme linked-immuno-electrotransfer" ) OR TS=( "enzyme linked-immuno-electro transfer" ) OR TS=( "enzyme linked-immunoelectro-transfer" ) OR TS=( "enzyme linked-immuno electro-transfer" ) OR TS=( "enzyme linked-immuno-electro-transfer" ) OR TS=( "enzyme linked-immune electrotransfer" ) OR TS=( "enzyme linked-immune-electrotransfer" ) OR TS=( "enzyme linked-immune-electro transfer" ) OR TS=( "enzyme linked-immune electro-transfer" ) OR TS=( "enzyme linked-immune-electro-transfer" ) OR TS=( "enzyme-linked-immunoelectrotransfer" ) OR TS=( "enzyme-linked-immunoelectro transfer" ) OR TS=( "enzyme-linked-immuno electrotransfer" ) OR TS=( "enzyme-linked-immuno-electrotransfer" ) OR TS=( "enzyme-linked-immuno-electro transfer" ) OR TS=( "enzyme-linked-immunoelectro-transfer" ) OR TS=( "enzyme-linked-immuno electro-transfer" ) OR TS=( "enzyme-linked-immuno-electro-transfer" ) OR TS=( "enzyme-linked-immune electrotransfer" ) OR TS=( "enzyme-linked-immune-electrotransfer" ) OR TS=( "enzyme-linked-immune-electro transfer" ) OR TS=( "enzyme-linked-immune electro-transfer" ) OR TS=( "enzyme-linked-immune-electro-transfer" ) OR TS=( immunoblot ) OR TS=( immunoblotting ) OR TS=( "immuno blot" ) OR TS=( "immuno blotting" ) OR TS=( immuneblot ) OR TS=( immuneblotting ) OR TS=( "immune blot" ) OR TS=( "immune blotting" ) OR TS=( electroimmunotransfer ) OR TS=( "electro immunotransfer" ) OR TS=( "electroimmuno transfer" ) OR TS=( "electro immuno transfer" ) OR TS=( "electro-immunotransfer" ) OR TS=( "electro-immuno transfer" ) OR TS=( "electro immuno-transfer" ) OR TS=( "electro-immuno-transfer" ) OR TS=( "electroimmunetransfer" ) OR TS=( "electro immunetransfer" ) OR TS=( "electroimmune transfer" ) OR TS=( "electro immune transfer" ) OR TS=( "electro-immunetransfer" ) OR TS=( "electro-immune transfer" ) OR TS=( "electro immune-transfer" ) OR TS=( "electro-immune-transfer" ) OR TS=( "western blot" ) OR TS=( "western blotting" ) OR TS=( "DOT blot" ) OR TS=( "DOT blotting" ) OR TS=( "EITB") |

| 12 | TS=("ELISA") OR TS=("EIA") OR TS=("enzyme linked immunosorbent") OR TS=("enzyme linked immuno sorbent") OR TS=("enzyme linked immuno-sorbent") OR TS=("enzyme linked immunesorbent") OR TS=("enzyme linked immune sorbent") OR TS=("enzyme linked immune-sorbent") OR TS=("enzyme-linked immunosorbent") OR TS=("enzyme-linked immuno sorbent") OR TS=("enzyme-linked immuno-sorbent") OR TS=("enzyme-linked immunesorbent") OR TS=("enzyme-linked immune sorbent") OR TS=("enzyme-linked immune-sorbent") OR TS=("enzyme linked-immunosorbent") OR TS=("enzyme linked-immuno sorbent") OR TS=("enzyme linked-immuno-sorbent") OR TS=("enzyme linked-immunesorbent") OR TS=("enzyme linked-immune sorbent") OR TS=("enzyme linked-immune-sorbent") OR TS=("enzyme-linked-immunosorbent") OR TS=("enzyme-linked-immuno sorbent") OR TS=("enzyme-linked-immuno-sorbent") OR TS=("enzyme-linked-immunesorbent") OR TS=("enzyme-linked-immune sorbent") OR TS=("enzyme-linked-immune-sorbent") OR TS=("enzyme-immuno assay") OR TS=("enzyme-immunoassay") OR TS=("enzyme-immuno-assay") OR TS=("enzyme-immune assay") OR TS=("enzyme-immune-assay") OR TS=("DOT ELISA") OR TS=("DOT-ELISA") OR TS=("Ag ELISA") OR TS=("Ag-ELISA") OR TS=("Ab ELISA") OR TS=("Ab-ELISA") |
| --- | --- |
| 13 | TS=(multiplex) OR TS=("multiple bead") OR TS=("multiple-bead") OR TS=("bead based") OR TS=("bead-based") |
| 14 | TS=("point-of-care test*") OR TS=("point of care test*") OR TS=("POC") |
| 15 | TS=("Immunologic* Test*") OR TS=(immunoassay) OR TS=("immuno assay") OR TS=("immuno-assay") OR TS=("immune assay") OR TS=("immune-assay") OR TS=(immunologic*) OR TS=(immunodiagnosis) OR TS=(immunodiagnostic*) OR TS=(immunodetect*) OR TS=("Serologic* Test*") OR TS=(serodiagnosis) OR TS=(serodiagnostic*) OR TS=("antigen assay") OR TS=("antigen-assay") OR TS=("antibody assay") OR TS=("antibody-assay") |
| 16 | TS=("diagnostic marker") OR TS=("diagnostic antigen") OR TS=("diagnostic antibody") OR TS=("diagnostic protein") OR TS=("diagnostic peptide") OR TS=("diagnostic reagent") OR TS=("immun* marker") OR TS=("immun* antigen") OR TS=("immun* antibody") OR TS=("immun* protein") OR TS=("immun* peptide") OR TS=("immun* reagent") OR TS=(biomarker) OR TS=(biosensor) OR TS=(mimotope) OR TS=("antigen diagnos*") OR TS=("antibody diagnos*") |
| 17 | #11 OR #12 OR #13 OR #14 OR #15 OR #16 |
| 18 | TS=(PCR) OR TS=(qPCR) OR TS=("polymerase chain reaction") OR TS=("polymerase-chain reaction") OR TS=("polymerase chain-reaction") OR TS=("polymerase-chain-reaction") |
| 19 | (#11 OR #12 OR #13 OR #14 OR #15 OR #16) NOT #18 |
| 20 | TS=(sensitiv*) OR TS=(specific*) OR TS=(predict*) OR TS=(evaluat*) OR TS=(accurate) OR TS=(accuracy) OR TS=(valid*) OR TS=(perform*) OR TS=("cross react*") OR TS=("cross-react*") OR TS=(utili*) OR TS=(applic*) |
| 21 | TS=(urine) OR TS=(urin*) OR TS=(serolog*) OR TS=(serum) OR TS=(sera) OR TS=(blood) OR TS=(plasma) OR TS=(antigen*) OR TS=(antibody) OR TS=(antibodies) |
| 22 | #10 AND #19 AND #20 AND #21 |

**PubMed**

| **#** | **Search terms** |
| --- | --- |
| 1 | ((("Neurocysticercosis"[MeSH Terms]) OR (neurocysticerc*[Title/Abstract])) OR (neurocisticerc*[Title/Abstract])) |
| 2 | (human cysticerc*[Title/Abstract]) OR (human cisticerc*[Title/Abstract]) |
| 3 | (("Cysticercosis"[MeSH Terms]) OR (cysticerc*[Title/Abstract])) OR (cisticerc*[Title/Abstract]) |
| 4 | (cyst[Title/Abstract]) OR (cystic lesion[Title/Abstract]) OR (cystical lesion[Title/Abstract]) OR (cestod*[Title/Abstract]) OR (metacestod*[Title/Abstract]) |
| 5 | ((("Taenia solium"[Mesh]) OR (taenia solium[Title/Abstract])) OR (tenia solium[Title/Abstract])) OR (T. solium[Title/Abstract]) |
| 6 | #4 AND #5 |
| 7 | #3 OR #6 |
| 8 | ((((((((((((((((((((((brain[Title/Abstract]) OR (cerebral[Title/Abstract])) OR (central nervous[Title/Abstract])) OR (central nervous system[Title/Abstract])) OR (CNS[Title/Abstract])) OR (neuro*[Title/Abstract])) OR (neural[Title/Abstract])) OR (medullary[Title/Abstract])) OR (intramedullary[Title/Abstract])) OR (intra-medullary[Title/Abstract])) OR (extramedullary[Title/Abstract])) OR (extra-medullary[Title/Abstract])) OR (ventricular[Title/Abstract])) OR (subarachnoid*[Title/Abstract])) OR (parenchymal[Title/Abstract])) OR (intraparenchymal[Title/Abstract])) OR (intra-parenchymal[Title/Abstract])) OR (extraparenchymal[Title/Abstract])) OR (extra-parenchymal[Title/Abstract])) OR (spinal[Title/Abstract])) OR (spine[Title/Abstract])) OR (racemose[Title/Abstract])) |
| 9 | #7 AND #8 |
| 10 | #1 OR #2 OR #9 |
| 11 | ((((((((((((((((((((((((((((((((((((((((((((((((((((((((((((((((((((((((((((((((("Immunoblotting"[MeSH Terms]) OR (enzyme linked immunoelectrotransfer[Title/Abstract])) OR (enzyme linked immunoelectro transfer[Title/Abstract])) OR (enzyme linked immuno electrotransfer[Title/Abstract])) OR (enzyme linked immuno-electrotransfer[Title/Abstract])) OR (enzyme linked immuno-electro transfer[Title/Abstract])) OR (enzyme linked immunoelectro-transfer[Title/Abstract])) OR (enzyme linked immuno electro-transfer[Title/Abstract])) OR (enzyme linked immuno-electro-transfer[Title/Abstract])) OR (enzyme linked immune electrotransfer[Title/Abstract])) OR (enzyme linked immune-electrotransfer[Title/Abstract])) OR (enzyme linked immune-electro transfer[Title/Abstract])) OR (enzyme linked immune electro-transfer[Title/Abstract])) OR (enzyme linked immune-electro-transfer[Title/Abstract])) OR (enzyme-linked immunoelectrotransfer[Title/Abstract])) OR (enzyme-linked immunoelectro transfer[Title/Abstract])) OR (enzyme-linked immuno electrotransfer[Title/Abstract])) OR (enzyme-linked immuno-electrotransfer[Title/Abstract])) OR (enzyme-linked immuno-electro transfer[Title/Abstract])) OR (enzyme-linked immunoelectro-transfer[Title/Abstract])) OR (enzyme-linked immuno electro-transfer[Title/Abstract])) OR (enzyme-linked immuno-electro-transfer[Title/Abstract])) OR (enzyme-linked immune electrotransfer[Title/Abstract])) OR (enzyme-linked immune-electrotransfer[Title/Abstract])) OR (enzyme-linked immune-electro transfer[Title/Abstract])) OR (enzyme-linked immune electro-transfer[Title/Abstract])) OR (enzyme-linked immune-electro-transfer[Title/Abstract])) OR (enzyme linked-immunoelectrotransfer[Title/Abstract])) OR (enzyme linked-immunoelectro transfer[Title/Abstract])) OR (enzyme linked-immuno electrotransfer[Title/Abstract])) OR (enzyme linked-immuno-electrotransfer[Title/Abstract])) OR (enzyme linked-immuno-electro transfer[Title/Abstract])) OR (enzyme linked-immunoelectro-transfer[Title/Abstract])) OR (enzyme linked-immuno electro-transfer[Title/Abstract])) OR (enzyme linked-immuno-electro-transfer[Title/Abstract])) OR (enzyme linked-immune electrotransfer[Title/Abstract])) OR (enzyme linked-immune-electrotransfer[Title/Abstract])) OR (enzyme linked-immune-electro transfer[Title/Abstract])) OR (enzyme linked-immune electro-transfer[Title/Abstract])) OR (enzyme linked-immune-electro-transfer[Title/Abstract])) OR (enzyme-linked-immunoelectrotransfer[Title/Abstract])) OR (enzyme-linked-immunoelectro transfer[Title/Abstract])) OR (enzyme-linked-immuno electrotransfer[Title/Abstract])) OR (enzyme-linked-immuno-electrotransfer[Title/Abstract])) OR (enzyme-linked-immuno-electro transfer[Title/Abstract])) OR (enzyme-linked-immunoelectro-transfer[Title/Abstract])) OR (enzyme-linked-immuno electro-transfer[Title/Abstract])) OR (enzyme-linked-immuno-electro-transfer[Title/Abstract])) OR (enzyme-linked-immune electrotransfer[Title/Abstract])) OR (enzyme-linked-immune-electrotransfer[Title/Abstract])) OR (enzyme-linked-immune-electro transfer[Title/Abstract])) OR (enzyme-linked-immune electro-transfer[Title/Abstract])) OR (enzyme-linked-immune-electro-transfer[Title/Abstract])) OR (immunoblot[Title/Abstract])) OR (immunoblotting[Title/Abstract])) OR (immuno blot[Title/Abstract])) OR (immuno blotting[Title/Abstract])) OR (immuneblot[Title/Abstract])) OR (immuneblotting[Title/Abstract])) OR (immune blot[Title/Abstract])) OR (immune blotting[Title/Abstract])) OR (electroimmunotransfer[Title/Abstract])) OR (electro immunotransfer[Title/Abstract])) OR (electroimmuno transfer[Title/Abstract])) OR (electro immuno transfer[Title/Abstract])) OR (electro-immunotransfer[Title/Abstract])) OR (electro-immuno transfer[Title/Abstract])) OR (electro immuno-transfer[Title/Abstract])) OR (electro-immuno-transfer[Title/Abstract])) OR (electroimmunetransfer[Title/Abstract])) OR (electro immunetransfer[Title/Abstract])) OR (electroimmune transfer[Title/Abstract])) OR (electro immune transfer[Title/Abstract])) OR (electro-immunetransfer[Title/Abstract])) OR (electro-immune transfer[Title/Abstract])) OR (electro immune-transfer[Title/Abstract])) OR (electro-immune-transfer[Title/Abstract])) OR (Western blot[Title/Abstract])) OR (western blotting[Title/Abstract])) OR (DOT blot[Title/Abstract])) OR (DOT blotting[Title/Abstract])) OR (EITB[Title/Abstract]) |
| 12 | ((((((((((((((((((((((((((((((((((((("Enzyme-Linked Immunosorbent Assay"[MeSH Terms]) OR (ELISA[Title/Abstract])) OR (EIA[Title/Abstract])) OR (enzyme linked immunosorbent[Title/Abstract])) OR (enzyme linked immuno sorbent[Title/Abstract])) OR (enzyme linked immuno-sorbent[Title/Abstract])) OR (enzyme linked immunesorbent[Title/Abstract])) OR (enzyme linked immune sorbent[Title/Abstract])) OR (enzyme linked immune-sorbent[Title/Abstract])) OR (enzyme-linked immunosorbent[Title/Abstract])) OR (enzyme-linked immuno sorbent[Title/Abstract])) OR (enzyme-linked immuno-sorbent[Title/Abstract])) OR (enzyme-linked immunesorbent[Title/Abstract])) OR (enzyme-linked immune sorbent[Title/Abstract])) OR (enzyme-linked immune-sorbent[Title/Abstract])) OR (enzyme linked-immunosorbent[Title/Abstract])) OR (enzyme linked-immuno sorbent[Title/Abstract])) OR (enzyme linked-immuno-sorbent[Title/Abstract])) OR (enzyme linked-immunesorbent[Title/Abstract])) OR (enzyme linked-immune sorbent[Title/Abstract])) OR (enzyme linked-immune-sorbent[Title/Abstract])) OR (enzyme-linked-immunosorbent[Title/Abstract])) OR (enzyme-linked-immuno sorbent[Title/Abstract])) OR (enzyme-linked-immuno-sorbent[Title/Abstract])) OR (enzyme-linked-immunesorbent[Title/Abstract])) OR (enzyme-linked-immune sorbent[Title/Abstract])) OR (enzyme-linked-immune-sorbent[Title/Abstract])) OR (enzyme-immuno assay[Title/Abstract])) OR (enzyme-immunoassay[Title/Abstract])) OR (enzyme-immuno-assay[Title/Abstract])) OR (enzyme-immune assay[Title/Abstract])) OR (enzyme-immune-assay[Title/Abstract])) OR (DOT ELISA[Title/Abstract])) OR (DOT-ELISA[Title/Abstract])) OR (Ag ELISA[Title/Abstract])) OR (Ag-ELISA[Title/Abstract])) OR (Ab ELISA[Title/Abstract])) OR (Ab-ELISA[Title/Abstract]) |
| 13 | ((((multiplex[Title/Abstract]) OR (multiple bead[Title/Abstract])) OR (multiple-bead[Title/Abstract])) OR (bead based[Title/Abstract])) OR (bead-based[Title/Abstract]) |
| 14 | ((("Point-of-Care Testing"[MeSH Terms]) OR (point-of-care test*[Title/Abstract])) OR (point of care test*[Title/Abstract])) OR (POC[Title/Abstract]) |
| 15 | (((((((((((((((("Immunologic Tests"[MeSH Terms]) OR (immunoassay[Title/Abstract])) OR (immuno assay[Title/Abstract])) OR (immuno-assay[Title/Abstract])) OR (immune assay[Title/Abstract])) OR (immune-assay[Title/Abstract])) OR (immunologic*[Title/Abstract])) OR (immunodiagnosis[Title/Abstract])) OR (immunodiagnostic*[Title/Abstract])) OR (immunodetect*[Title/Abstract])) OR ("Serologic Tests"[MeSH Terms])) OR (serodiagnosis[Title/Abstract])) OR (serodiagnostic*[Title/Abstract])) OR (antigen assay[Title/Abstract])) OR (antigen-assay[Title/Abstract])) OR (antibody assay[Title/Abstract])) OR (antibody-assay[Title/Abstract]) |
| 16 | ((((((((((((((((diagnostic marker[Title/Abstract]) OR (diagnostic antigen[Title/Abstract])) OR (diagnostic antibody[Title/Abstract])) OR (diagnostic protein[Title/Abstract])) OR (diagnostic peptide[Title/Abstract])) OR (diagnostic reagent[Title/Abstract])) OR (immun* marker[Title/Abstract])) OR (immun* antigen[Title/Abstract])) OR (immun* antibody[Title/Abstract])) OR (immun* protein[Title/Abstract])) OR (immun* peptide[Title/Abstract])) OR (immun* reagent[Title/Abstract])) OR (biomarker[Title/Abstract])) OR (biosensor[Title/Abstract])) OR (mimotope[Title/Abstract])) OR (antigen diagnos*[Title/Abstract])) OR (antibody diagnos*[Title/Abstract]) |
| 17 | #11 OR #12 OR #13 OR #14 OR #15 OR #16 |
| 18 | (((((PCR[Title/Abstract]) OR (qPCR[Title/Abstract])) OR (polymerase chain reaction[Title/Abstract])) OR (polymerase-chain reaction[Title/Abstract])) OR (polymerase chain-reaction[Title/Abstract])) OR (polymerase-chain-reaction[Title/Abstract]) |
| 19 | #17 NOT #18 |
| 20 | (((((((((((sensitiv*[Title/Abstract]) OR (specific*[Title/Abstract])) OR (predict*[Title/Abstract])) OR (evaluat*[Title/Abstract])) OR (accurate[Title/Abstract])) OR (accuracy[Title/Abstract])) OR (valid*[Title/Abstract])) OR (perform*[Title/Abstract])) OR (cross react*[Title/Abstract])) OR (cross-react*[Title/Abstract])) OR (utili*[Title/Abstract])) OR (applic*[Title/Abstract]) |
| 21 | (((((((((((urine[Title/Abstract]) OR (urin*[Title/Abstract])) OR (serolog*[Title/Abstract])) OR (serum[Title/Abstract])) OR (sera[Title/Abstract])) OR (blood[Title/Abstract])) OR (plasma[Title/Abstract])) ) OR (antigen*[Title/Abstract])) OR (antibody[Title/Abstract])) OR (antibodies[Title/Abstract]) |
| 22 | #10 AND #19 AND #20 AND #21 |
