# Supplementary material for: Accuracy of immunological tests on serum and urine for diagnosis of Taenia solium neurocysticercosis: A systematic review
Source: PLoS Negl Trop Dis. 2024 Nov 11;18(11):e0012643. doi: 10.1371/journal.pntd.0012643 (PMC11581404; doi:10.1371/journal.pntd.0012643)
Supplement: S1 Protocol — (PDF) [file pntd.0012643.s003.pdf]

A systematic review of accuracy of immunological tests and diagnostic markers for diagnosis of neurocysticercosis in serum and urine

To enable PROSPERO to focus on COVID-19 submissions, this registration record has undergone basic automated checks for eligibility and is published exactly as submitted. PROSPERO has never provided peer review, and usual checking by the PROSPERO team does not endorse content. Therefore, automatically published records should be treated as any other PROSPERO registration. Further detail is provided here.

Citation

Lisa Van Acker, Luz Toribio Salazar, Mkunde Chachage, Hang Zeng, Brecht Devleesschauwer, Hector H. García, Sarah Gabriël. A systematic review of accuracy of immunological tests and diagnostic markers for diagnosis of neurocysticercosis in serum and urine. PROSPERO 2023 CRD42023440930 Available from: https://www.crd.york.ac.uk/prospero/display_record.php?ID=CRD42023440930

Review question

How do different immunological tests, and utilized diagnostic markers, on biological specimens serum/urine for diagnosis of neurocysticercosis perform regarding accuracy? What is the performed accuracy for diagnosis of neurocysticercosis with different cyst amount, location and biological phase?

Searches

Following electronic bibliographic databases will be searched: EMBASE, PubMed, Scopus, Web of Science, with no restrictions regarding language. There will be no restriction regarding publication date. Clinical trial registries or grey literature databases will not be consulted. Literature search strategies will be developed using both medical subject headings (MeSH) and text words related to immunological diagnosis of neurocysticercosis. First, a PubMed search strategy will be developed. After the PubMed search is finalized, the search strategy will be adapted to the syntax and subject headings of the other databases. Additional references will be sought via backward snowballing. Searches will be re-run just before final analyses, to include additional studies.

Types of study to be included

Immunoassay diagnostic studies (cohort studies/ case-control studies/ cross-sectional studies) will be enrolled, specifically diagnostic accuracy studies. Case studies, (conference) abstracts, poster presentations, letters, comments, editorials, reports, thesis papers, methodologies of a study, and reviews will be excluded. Records without accessible full-text will be excluded.

Condition or domain being studied

Neurocysticercosis, caused by human (accidental intermediate host) Taenia solium infection; immunological diagnosis.

Participants/population

Patient population: general human population, with no restrictions on demographics or clinical characteristics.

Reference standard: the used reference standard to establish diagnosis of neurocysticercosis will be CT, MRI, or surgery/biopsy. Humans with diagnosis of neurocysticercosis (with no restrictions on phase, amount, or localization of

cysts), confirmed via neuroimaging (CT and/or MRI) and/or surgery/biopsy, will be included in the NCC group.

Control: control groups will consist of humans without neurocysticercosis (suggested/presumed/confirmed). May be healthy controls, people with seizures but without neurocysticercosis, or humans with T. solium taeniasis or other infectious diseases.

Intervention(s), exposure(s)

Concerning immunological tests (index tests) on biological specimens serum/urine, for detection of antibodies/antigens in diagnosis of neurocysticercosis. Also records evaluating diagnostic markers in diagnosis of neurocysticercosis, will be included. Evaluation of a test/marker implies test characteristics, operational characteristics, and performance including accuracy. The accuracy can be represented as accuracy, Se, Sp, PPV, NPV, OR, LR+, LR- , ROC-curve, or AUC. For a record to be included, accuracy is either mentioned in the record, or can be calculated with available data.

All samples that are used to determine sensitivity, must originate from humans with neurocysticercosis confirmed via neuroimaging or surgery/biopsy, if not, the record is excluded. All samples that are used to determine specificity, must originate from humans without neurocysticercosis, if not, the record is excluded. Records with the main focus of determining seroprevalence or clinical characteristics/profile by use of an immunological test, are excluded, unless evaluation of a test is mentioned as an aim of the study and obtained values for accuracy are specifically mentioned in the abstract.

Furthermore, studies on tests/markers co-detecting T. solium taeniasis, or a different infectious disease, will be included. Records concerning PCR methods, or records using only CSF as specimen, will be excluded.

Comparator(s)/control

Humans from the general human population, with no restrictions on demographics or clinical characteristics, without neurocysticercosis (suggested/presumed/confirmed). May be healthy controls, people with seizures but without neurocysticercosis, or humans with T. solium taeniasis or other infectious diseases.

Context

Taenia solium is a foodborne zoonotic tapeworm, a neglected tropical disease, endemic in middle- and low- income countries of sub-Saharan Africa, South- and South-East Asia, and Latin-America. Imported cases are sporadically seen in non-endemic high-income countries. In this review, we will not differentiate between resource-limited or resource-abundant areas, or between any types of healthcare facilities, as the aim is to provide an overview of all immunological tests/markers.

Currently, neuroimaging techniques (e.g. CT/MRI) are regarded as the gold standard for diagnosis of neurocysticercosis. However, neuroimaging modalities are expensive, require trained staff, and are often unavailable or inaccessible, certainly in endemic countries. Immunological methods could pose an alternative to neuroimaging, however, knowledge is lacking on characteristics and performance of these tests.

Regarding systematic reviews on immunological diagnosis of neurocysticercosis, Cardona-Arias et al., 2017 has published a meta-analysis on this matter, however, more recent records are not yet included in this review, and the review does not hold into account the performance of tests by amount, localisation and phases of cysticerci. The protocol for another review [CRD42019144897], similar to our ongoing review, has been registered by Carpio et al. However, registered in 2019, no following publication can be allocated. Furthermore, this review does not mention inclusion of records on diagnostic markers, and mentions fewer data collection on test characteristics. Additionally, in our ongoing review we will not include studies on CSF, deeming results of the review more applicable to conditions where invasive techniques are not conceivable, such as endemic areas.

Main outcome(s)

Accuracy of the evaluated immunological test, or immunologically assessed diagnostic marker.

Measures of effect

The primary accuracy measures will be sensitivity and specificity.

Additional outcome(s)

Further assessment and subdivision of accuracy will be made according to differences in cyst location (i.e. parenchymal/extra-parenchymal), amount (number), and phase (active, transition, calcified) of patients diagnosed with confirmed neurocysticercosis (NCC group).

Measures of effect

The primary accuracy measure will be sensitivity.

Data extraction (selection and coding)

The results from the four search engines will be merged in EndNote, and duplicates will be removed. Next, records will be screened for eligibility in Rayyan, using title and abstract, identifying studies that potentially meet inclusion criteria or are excluded via exclusion criteria outlined above. Likewise, full-texts of potentially eligible records will further be screened. Screening will be done by 2 independent authors (LVA, LST), and data collection by 1 author (LVA). In case of records with full-text not in English, screening and data collection will be conducted by 1 author (LST/HZ).

Data will be collected on:

Concerning study characteristics:

Author, title, year of publication, journal, language of full-text, study type, country where the study is performed.

Concerning study participants:

Information regarding the selected groups, amount of selected participants per group, gender and age, clinical symptoms of participants, reference standard, and location, amount, and phase of cysts in patients of the NCC group.

Concerning collected samples:

Information regarding sample type (i.e. serum/urine), sample volume, procurement, dilution, storage, collection site (e.g. trial/database).

Concerning test/marker evaluation:

Information regarding test identification will be gathered, i.e. type and name of the immunological test/technique, laboratory and country of development, diagnostic marker used (including type , name, methodology of procurement/synthetisation and preparation), and analyte.

Further extracted data will include:

a) test characteristics

i. intended use (i.e. screening/identification/follow-up) and setting (i.e. specification of clinical/field setting)

ii. co-detection

b) operational characteristics

i. methodology of test execution (from sample handling to test result; including number of steps, time needed, and equipment needs)

ii. nature of the test result (i.e. quantitative/semi-quantitative/qualitative)

iii. difficulty of test execution/interpretation

c) test performance

i. cut-off value

ii. accuracy

iii. cross-reactivity

d) phase of diagnostic test development

e) test validity

i. methodology of validation

f) commercial availability

Risk of bias (quality) assessment

Risk of bias assessment will be conducted by 1 author (LVA, or in case of records not in English LST/HZ). To evaluate the risk of bias, and specifically verification bias in studies primarily assessing accuracy, the QUADAS-2 tool will be used.

The following will be assessed, based on the QUADAS-2 tool:

Could the patient selection have introduced bias?

Was a consecutive or random sample of patients enrolled?

Was a case–control design avoided?

Did the study avoid inappropriate exclusions?

Could the conduct or interpretation of the index test have introduced bias?

Were the index test results interpreted without knowledge of the results of the reference standard?

If a threshold was used, was it prespecified?

Could the reference standard, its conduct, or its interpretation have introduced bias?

Is the reference standard likely to correctly classify the target condition?

Were the reference standard results interpreted without knowledge of the results of the index test?

Could the patient flow have introduced bias?

Was there an appropriate interval between the index test and reference standard?

Did all patients receive the same reference standard?

Were all patients included in the analysis?

Strategy for data synthesis

Following obstacles could be encountered during data collection and synthesis, and will be dealt with as described: (i) handling of indeterminate data: the main author of the record will be contacted in the case of indeterminate data crucial for complete data synthesis. If there is no response received from the author, this data will not be included in data synthesis. (ii) handling of different reference standards: we will assume that all participants of the NCC group that have been diagnosed with neurocysticercosis via at least one of the mentioned reference standards can be categorized as confirmed cases of neurocysticercosis. In data collection and synthesis, no distinction will be made between data originating from participants diagnosed via a different reference test.

Should records be conceptually comparable and use the same measures of diagnostic accuracy, a meta-analysis will be

conducted. Data on diagnostic accuracy measures will be extracted from the eligible studies. Results from studies using the same diagnostic method and that are conceptually comparable will be plotted visually using a forest plot. Using a random effects model, heterogeneity will be assessed by calculating the I^2. Values of $I^2 > 0.30$ will be deemed important, and values of $I^2 > 0.50$ highly important. The meta-analysis will be carried out using R as software.

Should a meta-analysis not be applicable, we will synthesize the data on diagnostic accuracy narratively. The range of estimates concerning accuracy will be presented for each diagnostic test/marker (if this data is available). An ROC curve will be used to present the performance of an immunological test. All information on other outcomes, and other data extracted, will be synthesized narratively.

Analysis of subgroups or subsets

NA

Contact details for further information

Lisa Van Acker

lisa.vanacker@ugent.be

Organisational affiliation of the review

Ghent University

https://www.ugent.be/

Review team members and their organisational affiliations [2 changes]

Ms Lisa Van Acker. Ghent University

Ms Luz Toribio Salazar. Universidad Peruana Cayetano Heredia, St George's University of London

Dr Mkunde Chachage. University of Dar es Salaam

Dr Hang Zeng. Xihua University

Professor Brecht Devleesschauwer. Sciensano, Ghent University

Professor Hector H. García. Universidad Peruana Cayetano Heredia, Johns Hopkins University

Professor Sarah Gabriël. Ghent University

Type and method of review

Diagnostic, Systematic review

Anticipated or actual start date

08 May 2023

Anticipated completion date [1 change]

31 May 2024

Funding sources/sponsors

This systematic review is situated within NeuroSolve, a Horizon Europe project, EDCTP funded.

Conflicts of interest

Language

English

Country

Belgium, China, Peru

Stage of review [1 change]

Review Completed not published

Subject index terms status

Subject indexing assigned by CRD

Subject index terms

Animals; Body Fluids; Humans; Immunologic Tests; Neurocysticercosis; Taenia solium

Date of registration in PROSPERO

17 July 2023

Date of first submission

06 July 2023

Stage of review at time of this submission [2 changes]

**Stage**	**Started**	**Completed**
Preliminary searches	Yes	Yes
Piloting of the study selection process	Yes	Yes
Formal screening of search results against eligibility criteria	Yes	Yes
Data extraction	Yes	Yes
Risk of bias (quality) assessment	Yes	Yes
Data analysis	Yes	Yes

Revision note

Updated author affiliations, and updated review status.

The record owner confirms that the information they have supplied for this submission is accurate and complete and they understand that deliberate provision of inaccurate information or omission of data may be construed as scientific misconduct.

The record owner confirms that they will update the status of the review when it is completed and will add publication details in due course.

Versions

17 July 2023

17 July 2023

25 March 2024

17 April 2024

17 June 2024
