# Supplementary material for: Accuracy of immunological tests on serum and urine for diagnosis of Taenia solium neurocysticercosis: A systematic review
Source: PLoS Negl Trop Dis. 2024 Nov 11;18(11):e0012643. doi: 10.1371/journal.pntd.0012643 (PMC11581404; doi:10.1371/journal.pntd.0012643)
Supplement: S1 Text — (DOCX) [file pntd.0012643.s004.docx]

**S1 Text. Adapted signalling questions for QUADAS-2 Risk of Bias assessment.**

**DOMAIN 1 : Patient selection**

*Notes*

**Q1**: Was a consecutive or random sample of patients enrolled?

Yes / No / Unclear

**Q2**: Was a case-control design avoided (i.e. Were individuals not divided into a case group or a control group before index test performance?)

Yes / No / Unclear

**Q3:** Did the study avoid inappropriate exclusion of patients?

**Q3.1:** Did the study avoid deliberate exclusion of patients with taeniasis or other infections?

Yes / No / Unclear
**Q3.2:** Did the study avoid deliberate exclusion of patients with a specific type of cysts (regarding location/stage/amount of cysts)?

Yes / No / Unclear

**Q4**: Were all control individuals and individuals with other infections confirmed to not have NCC (target condition) via the reference standard?

Yes / No / Unclear / NA

**DOMAIN 2: Index test(s)**

*Notes*

**Q5:** Were all samples of patients tested via the same method (1 index test or the same combination of multiple index tests)?
 Yes / No / Unclear

Did it occur that multiple index tests were used to test the same sample?

No.

Unclear.
 Yes. **Q6:** Were the results of the index tests interpreted blind to each other?

Yes / No / Unclear / NA

| Used index test (test / diagnostic marker) | **Q7**: Were the index test results interpreted without knowledge of the results of the reference standard (i.e. blinding to the reference standard results)? | Was a threshold used? No/Not indicated for this test.  Yes/Most likely. **Q8**: Was the value of the threshold or the method of threshold calculation prespecified and exclusive? | **Q9:**  Is uncontradictory data available (+included for analysis) on all performed index tests for…: | |
| --- | --- | --- | --- | --- |
|  |  |  | **Q9.1: …**patients of the NCC Group? | **Q9.2: …**patients of the Control Group/Other Infections Group (as applicable)? |
|  | Yes / No / Unclear | Yes / No / Unclear / NA | Yes / No / Unclear | Yes / No / Unclear / NA |
|  | Yes / No / Unclear | Yes / No / Unclear / NA | Yes / No / Unclear | Yes / No / Unclear / NA |
|  | Yes / No / Unclear | Yes / No / Unclear / NA | Yes / No / Unclear | Yes / No / Unclear / NA |
|  | Yes / No / Unclear | Yes / No / Unclear / NA | Yes / No / Unclear | Yes / No / Unclear / NA |
|  | Yes / No / Unclear | Yes / No / Unclear / NA | Yes / No / Unclear | Yes / No / Unclear / NA |
|  | Yes / No / Unclear | Yes / No / Unclear / NA | Yes / No / Unclear | Yes / No / Unclear / NA |
|  | Yes / No / Unclear | Yes / No / Unclear / NA | Yes / No / Unclear | Yes / No / Unclear / NA |
|  | Yes / No / Unclear | Yes / No / Unclear / NA | Yes / No / Unclear | Yes / No / Unclear / NA |
|  | Yes / No / Unclear | Yes / No / Unclear / NA | Yes / No / Unclear | Yes / No / Unclear / NA |
|  | Yes / No / Unclear | Yes / No / Unclear / NA | Yes / No / Unclear | Yes / No / Unclear / NA |
|  | Yes / No / Unclear | Yes / No / Unclear / NA | Yes / No / Unclear | Yes / No / Unclear / NA |
|  | Yes / No / Unclear | Yes / No / Unclear / NA | Yes / No / Unclear | Yes / No / Unclear / NA |

**DOMAIN 3: Reference standard(s)**

*Notes*

**Q10:** Were all samples of patients of the NCC Group tested via the same method (1 reference standard or the same combination of multiple reference standards)?
 Yes / No / Unclear

Did it occur that multiple reference standards were used to test the same sample?

No.
Yes. **Q11:** Were the results of the reference standards interpreted blind to each other?

Yes / No / Unclear / NA

| Used reference standard | **Q12**: Is the reference standard likely to correctly classify the target condition? | **Q13**: Were the reference standard results interpreted without knowledge of the results of the index test (i.e. blinding to the index test results)? |
| --- | --- | --- |
|  | Yes / No / Unclear | Yes / No / Unclear |
|  | Yes / No / Unclear | Yes / No / Unclear |
|  | Yes / No / Unclear | Yes / No / Unclear |

**DOMAIN 4: Flow and timing**

*Notes*

**Q14**: Was there an appropriate interval between sample procurement (for index test) and index test (depending on sample type, volume and storage method)?

Yes / No / Unclear

**Q15**: Was there an appropriate interval of maximum 2 months between sample procurement (for index test) and reference standard, without commencement of treatment within this interval?

Yes / No / Unclear

**Q16**: Was the choice of used reference standard independent of the results of the index test?

Yes / No / Unclear
