# Supplementary material for: Accuracy of immunological tests on serum and urine for diagnosis of Taenia solium neurocysticercosis: A systematic review
Source: PLoS Negl Trop Dis. 2024 Nov 11;18(11):e0012643. doi: 10.1371/journal.pntd.0012643 (PMC11581404; doi:10.1371/journal.pntd.0012643)
Supplement: S1 Table — (DOCX) [file pntd.0012643.s005.docx]

**S1 Table. Quality Assessment (QUADAS-2) summary of included studies: Risk of Bias.**

|  | Q1 | Q2 | Q3.1 | Q3.2 | Q4 | Q5 | Q6 | Q7 | Q8 | Q9.1 | Q9.2 | Q10 | Q11 | Q12 | Q13 | Q14 | Q15 | Q16 |
| --- | --- | --- | --- | --- | --- | --- | --- | --- | --- | --- | --- | --- | --- | --- | --- | --- | --- | --- |
| Agrawal 2017 [1] | Unclear | No | Unclear | Yes | Unclear | Yes | NA | Unclear | Yes | Yes | Yes | Unclear | Unclear | Yes | Yes | Unclear | Unclear | Yes |
| Agudelo 2005 [2] | Unclear | No | Yes | Unclear | No | Yes | Unclear | Unclear | Unclear | Yes | Yes | Yes | Unclear | Yes | Yes | Unclear | Unclear | Yes |
| Aguilar-Rebolledo 2002 [3] | Unclear | No | Unclear | Yes | Yes | Yes | NA | Unclear | Yes | Yes | Yes | No | Unclear | Yes | Yes | Unclear | Unclear | Yes |
| Ahn 2016 [4] | Unclear | No | Yes | Yes | No | Yes | NA | Unclear | NA | Yes | Yes | Unclear | Unclear | Yes | Yes | Unclear | Unclear | Yes |
| Anadure 2020 [5] | Unclear | No | Unclear | Yes | Yes | Yes | NA | Unclear | Unclear | Yes | Yes | Yes | NA | Yes | Yes | Unclear | Unclear | Yes |
| Arora 2020 [6] | Unclear | No | Unclear | Yes | No | No | Yes | Yes | Yes | Yes | Unclear | Yes | NA | Yes | Yes | Unclear | Unclear | Yes |
| Arruda 2005 [7] | Yes | No | Yes | Yes | No | Yes | No | Unclear | Yes | Yes | Yes | No | Unclear | Yes | Yes | Unclear | Unclear | Yes |
| Arruda 2006 [8] | Yes | No | Yes | Unclear | No | Yes | No | Unclear | Yes | Yes | Yes | No | Unclear | Yes | Yes | Unclear | Unclear | Yes |
| Arthi 2021 [9] | Unclear | No | Unclear | Yes | NA | Yes | NA | Unclear | Yes | Yes | NA | Yes | NA | Yes | Yes | Unclear | Unclear | Yes |
| Astudillo 2019 [10] | Unclear | No | Yes | Unclear | Yes | Yes | Unclear | Unclear | Yes | Yes | Yes | Yes | Unclear | Yes | Yes | Unclear | Unclear | Yes |
| Atluri 2009a [11] | Unclear | No | Yes | Yes | No | Yes | Unclear | Unclear | Yes | Yes | Yes | No | Unclear | Yes | Yes | Unclear | Unclear | Yes |
| Atluri 2009b [12] | Unclear | No | Yes | Yes | No | Yes | No | Unclear | Yes | Yes | Yes | No | Unclear | Yes | Yes | Unclear | Unclear | Yes |
| Atluri 2011 [13] | Unclear | No | Yes | Yes | No | Yes | NA | Unclear | Yes | Yes | Yes | No | Unclear | Yes | Yes | Unclear | Unclear | Yes |
| Ayala-Sulca 2015 [14] | No | No | Yes | Unclear | Yes | Yes | Unclear | Unclear | Yes | Yes | Yes | Yes | Unclear | Yes | Yes | Unclear | Unclear | Yes |
| Bae 2008 [15] | Unclear | No | Yes | Yes | No | Yes | Unclear | Unclear | Yes | Yes | Yes | No | Unclear | Yes | Yes | Unclear | Unclear | Yes |
| Bae 2014 [16] | Unclear | No | Yes | No | No | Yes | NA | Unclear | NA | Yes | Yes | Unclear | Unclear | Yes | Yes | Unclear | Unclear | Yes |
| Baily 1988 [17] | Unclear | No | Yes | Yes | No | No | Unclear | Unclear | Yes | No | No | No | Unclear | Yes | Yes | Unclear | Unclear | Yes |
| Barcelos 2005 [18] | Unclear | No | Unclear | Yes | Yes | Yes | Unclear | Unclear | Yes | Yes | Yes | Unclear | Unclear | Yes | Yes | Unclear | Unclear | Yes |
| Barcelos 2007 [19] | Unclear | No | Unclear | Yes | Yes | Yes | NA | Unclear | Yes | Yes | Yes | Unclear | Unclear | Yes | Yes | Unclear | Unclear | Yes |
| Biswas 2004 [20] | Unclear | No | Yes | Yes | No | Yes | Unclear | Yes | NA | Yes | Yes | No | Unclear | Yes | Yes | Unclear | Unclear | Yes |
| Brizzi 2016 [21] | Unclear | Yes | Yes | Yes | NA | Yes | Unclear | Unclear | Yes | Unclear | NA | Yes | NA | Yes | Yes | Unclear | Unclear | Yes |
| Bueno 2000a [22] | Unclear | No | Yes | Yes | No | No | No | Unclear | Yes | Yes | Yes | No | Unclear | Yes | Yes | Unclear | Unclear | Yes |
| Bueno 2000b [23] | Unclear | No | Yes | Yes | No | Yes | Unclear | Unclear | Yes | Yes | Yes | No | Unclear | Yes | Yes | Unclear | Unclear | Yes |
| Bueno 2005 [24] | Unclear | No | Yes | Yes | No | Yes | Unclear | Unclear | Yes | No | Yes | No | NA | Yes | Yes | Unclear | Unclear | Yes |
| Carod 2012 [25] | Unclear | No | Yes | Unclear | No | Yes | Unclear | Unclear | Yes | Yes | Yes | Yes | NA | Yes | Yes | Unclear | Unclear | Yes |
| Carrara 2020 [26] | Unclear | No | Yes | Yes | No | Yes | NA | Unclear | Yes | Yes | Yes | Unclear | Unclear | Yes | Yes | Unclear | Unclear | Yes |
| Castillo 2009 [27] | Yes | No | Yes | Yes | No | Yes | NA | Unclear | Yes | Yes | Yes | No | NA | Yes | Yes | Unclear | Yes | Yes |
| Castillo 2023 [28] | Yes | No | Unclear | No | NA | Yes | Yes | Yes | Yes | Yes | NA | No | NA | Yes | Yes | Unclear | Unclear | Yes |
|  | Q1 | Q2 | Q3.1 | Q3.2 | Q4 | Q5 | Q6 | Q7 | Q8 | Q9.1 | Q9.2 | Q10 | Q11 | Q12 | Q13 | Q14 | Q15 | Q16 |
| Chang 1988 [29] | Yes | Yes | Yes | Yes | Yes | Yes | NA | Unclear | Yes | Yes | Yes | No | Yes | Yes | Yes | Unclear | Unclear | Yes |
| Chuang 2017 [30] | Unclear | No | Yes | Unclear | No | Yes | NA | Unclear | Yes | Yes | Yes | No | Unclear | Yes | Yes | Unclear | Unclear | Yes |
| Cho 1986 [31] | Unclear | No | Yes | Yes | No | Yes | NA | Unclear | No | Yes | Yes | No | NA | Yes | Yes | Unclear | Unclear | Yes |
| Chung 1999 [32] | Unclear | No | Yes | Yes | No | Yes | NA | Unclear | NA | Yes | Yes | No | Unclear | Yes | Yes | Unclear | Unclear | Yes |
| Corona 1986 [33] | Unclear | No | Unclear | Yes | No | Yes | NA | Unclear | Yes | Yes | Yes | No | NA | Yes | Yes | Unclear | No | Yes |
| Corstjens 2014 [34] | Unclear | No | Yes | No | No | Yes | NA | Yes | No | Yes | Yes | No | NA | Yes | Yes | Unclear | Unclear | Yes |
| da Silva 2006 [35] | Unclear | No | Yes | Yes | No | Yes | NA | Unclear | Yes | Yes | Yes | Unclear | Unclear | Yes | Yes | Unclear | Unclear | Yes |
| da Silva 2020 [36] | Unclear | No | Yes | Yes | No | Yes | NA | Unclear | Yes | Yes | Yes | Unclear | Unclear | Yes | Yes | Unclear | Unclear | Yes |
| da Silva Ribeiro 2010a [37] | Unclear | No | Yes | Yes | No | Yes | Unclear | Unclear | Yes | Yes | Yes | Yes | NA | Yes | Yes | Unclear | Unclear | Yes |
| da Silva Ribeiro 2010b [38] | Unclear | No | Yes | Yes | No | Yes | Unclear | Unclear | Yes | Yes | Yes | Unclear | Unclear | Yes | Yes | Unclear | Unclear | Yes |
| Davelois 2016 [39] | Unclear | No | Yes | Unclear | Yes | Yes | Unclear | Unclear | Yes | Yes | Yes | Yes | Unclear | Yes | Yes | Unclear | Unclear | Yes |
| De Macedo 2002 [40] | Unclear | No | Yes | Unclear | Unclear | Yes | Unclear | Unclear | Yes | Yes | Yes | Yes | Unclear | Yes | Yes | Unclear | Unclear | Yes |
| Dermauw 2018 [41] | Yes | Yes | Yes | Yes | Yes | No | Unclear | Unclear | NA | Yes | Yes | Yes | NA | Yes | Unclear | Unclear | Unclear | Yes |
| Diaz 1992 [42] | Unclear | No | Yes | Unclear | No | Yes | Yes | Yes | Yes | Yes | Yes | No | Unclear | Yes | Yes | Unclear | Unclear | Yes |
| Eom 1988 [43] | Unclear | No | Yes | Unclear | No | Yes | Unclear | Unclear | Yes | Yes | Yes | No | Unclear | Yes | Yes | Unclear | Unclear | Yes |
| Espinoza 1986 [44] | Unclear | No | Yes | Yes | No | Yes | Unclear | Unclear | Yes | No | Yes | Yes | NA | Yes | Yes | Unclear | No | Yes |
| Espíndola 2005 [45] | Unclear | No | Yes | Unclear | No | Yes | NA | Unclear | Yes | Yes | Yes | No | Unclear | Yes | Yes | Unclear | Unclear | Yes |
| Feldman 1990 [46] | Unclear | No | Unclear | Yes | No | No | Unclear | Unclear | Yes | Yes | No | No | NA | Yes | Yes | Unclear | No | Yes |
| Ferrer 2002 [47] | Unclear | No | No | Unclear | NA | Yes | Unclear | Unclear | Yes | Yes | NA | No | NA | Yes | Yes | Unclear | Unclear | Yes |
| Ferrer 2003 [48] | Unclear | No | Yes | No | No | Yes | Unclear | Unclear | Yes | Yes | Yes | No | NA | Yes | Yes | Unclear | Unclear | Yes |
| Ferrer 2005a [49] | Unclear | No | Yes | Yes | No | Yes | Unclear | Unclear | Yes | Yes | Yes | No | NA | Yes | Yes | Unclear | Unclear | Yes |
| Ferrer 2005b [50] | Unclear | No | Yes | Yes | No | Yes | Unclear | Unclear | Yes | No | Yes | No | NA | Yes | Yes | Unclear | Unclear | Yes |
| Ferrer 2007a [51] | Unclear | No | Yes | Yes | No | Yes | Unclear | Unclear | Yes | Yes | Yes | No | Unclear | Yes | Yes | Unclear | Unclear | Yes |
| Ferrer 2007b [52] | Unclear | No | Yes | Yes | No | Yes | Unclear | Unclear | Yes | Yes | Yes | No | NA | Yes | Yes | Unclear | Unclear | Yes |
| Ferrer 2009 [53] | Unclear | No | Yes | Yes | No | Yes | Unclear | Unclear | No | Yes | Yes | No | NA | Yes | Yes | Unclear | Unclear | Yes |
| Ferrer 2012 [54] | Unclear | No | Yes | Yes | No | Yes | Unclear | Unclear | Yes | Yes | Yes | No | Unclear | Yes | Yes | Unclear | Unclear | Yes |
| Fleury 2001 [55] | Unclear | No | Unclear | Yes | Yes | No | Unclear | Unclear | Yes | Unclear | Unclear | No | Unclear | Yes | Yes | Unclear | Unclear | Yes |
| Fleury 2007 [56] | Unclear | No | Yes | Yes | Yes | No | Unclear | Yes | Yes | Yes | Yes | Yes | Unclear | Yes | Yes | Unclear | Unclear | Yes |
| Flisser 1980 [57] | Unclear | No | Unclear | Unclear | No | Yes | NA | Unclear | Yes | Yes | Yes | Yes | NA | Yes | Yes | Unclear | Unclear | Yes |
| Foyaca-Sibat 2009 [58] | Yes | Yes | Unclear | Yes | Yes | No | Unclear | Unclear | Yes | Yes | Yes | Yes | NA | Yes | Yes | Unclear | Unclear | Yes |
|  | Q1 | Q2 | Q3.1 | Q3.2 | Q4 | Q5 | Q6 | Q7 | Q8 | Q9.1 | Q9.2 | Q10 | Q11 | Q12 | Q13 | Q14 | Q15 | Q16 |
| Gabriël 2012 [59] | Unclear | No | Yes | Yes | Yes | Yes | NA | Yes | No | Yes | Yes | Yes | NA | Yes | Yes | No | Unclear | Yes |
| Garcia 2000 [60] | Unclear | No | Unclear | No | No | Yes | NA | Unclear | Yes | Yes | Yes | Yes | NA | Yes | Yes | Unclear | Unclear | Yes |
| Garcia 2002 [61] | Unclear | No | Unclear | No | NA | Yes | NA | Unclear | Yes | Yes | NA | Yes | NA | Yes | Yes | Unclear | Unclear | Yes |
| Garcia 2018 [62] | Unclear | No | Yes | No | No | Yes | Unclear | Unclear | Yes | Yes | Yes | Unclear | Unclear | Unclear | Yes | Unclear | Unclear | Yes |
| Gekeler 2002 [63] | Unclear | No | Yes | Unclear | No | Yes | Yes | Yes | No | Yes | Yes | No | Unclear | Yes | Yes | No | Unclear | Yes |
| Gonçalves 2010 [64] | Unclear | No | Yes | Yes | No | Yes | Unclear | Unclear | Yes | Yes | Yes | Unclear | Unclear | Yes | Yes | Unclear | Unclear | Yes |
| González 2010 [65] | Unclear | No | Unclear | Unclear | No | Yes | Unclear | Unclear | Yes | Yes | Yes | Yes | Unclear | Yes | Yes | Unclear | Unclear | Yes |
| Greene 1999 [66] | Unclear | No | Yes | Unclear | No | Yes | Unclear | Unclear | NA | Yes | Yes | No | Unclear | Yes | Yes | Unclear | Unclear | Yes |
| Hancock 2006 [67] | Unclear | No | Yes | Yes | NA | Yes | NA | Unclear | NA | Yes | Yes | No | NA | Yes | Yes | Unclear | Unclear | Yes |
| Handali 2010a [68] | Unclear | No | Yes | Yes | No | Yes | No | Yes | NA | Yes | Yes | No | NA | Yes | Yes | Unclear | Unclear | Yes |
| Handali 2010b [69] | Unclear | No | Yes | No | No | Yes | NA | Unclear | No | Yes | Yes | No | NA | Yes | Yes | Unclear | Unclear | Yes |
| Hell 2009 [70] | Unclear | No | Yes | Unclear | No | Unclear | Yes | Unclear | Yes | Yes | No | Unclear | Unclear | Yes | Yes | No | Unclear | Yes |
| Hernández 2019 [71] | Unclear | No | Yes | Yes | No | No | Unclear | Unclear | Yes | Unclear | Yes | Unclear | Unclear | Yes | Yes | Unclear | Unclear | Yes |
| Hernández-González 2017 [72] | Unclear | No | Yes | Yes | No | No | No | Unclear | Yes | Yes | Unclear | No | NA | Yes | Yes | Unclear | Unclear | Yes |
| Hernández-González 2022 [73] | Unclear | No | Yes | Yes | No | Yes | Unclear | Unclear | Yes | No | No | Unclear | Unclear | Yes | Yes | Unclear | Unclear | Yes |
| Hubert 1999 [74] | Unclear | No | Yes | Unclear | No | Yes | NA | Unclear | Yes | Yes | Yes | Yes | NA | Yes | Yes | Unclear | Unclear | Yes |
| Husain 2001 [75] | Unclear | No | Yes | No | Yes | Yes | NA | Yes | Yes | Yes | Yes | Unclear | Unclear | Yes | Yes | Unclear | Unclear | Yes |
| Intapan 2008 [76] | Unclear | No | Yes | Yes | No | Yes | Unclear | Unclear | Yes | Yes | Unclear | Unclear | Unclear | Yes | Unclear | Unclear | Unclear | Yes |
| Ishida 2003 [77] | Unclear | No | Yes | Unclear | No | No | Unclear | Unclear | Yes | Yes | Yes | Yes | NA | Yes | Yes | Unclear | Unclear | Yes |
| Ishida 2006 [78] | Unclear | No | Unclear | Yes | No | Yes | Unclear | Unclear | Yes | Yes | Yes | Yes | NA | Yes | Yes | Unclear | Unclear | Yes |
| Iudici 2007 [79] | Unclear | No | Yes | Unclear | No | Yes | Unclear | Unclear | Yes | Yes | Yes | Yes | NA | Yes | Yes | Unclear | Unclear | Yes |
| Jiménez 2022 [80] | Unclear | No | Yes | Yes | No | Yes | Unclear | Unclear | NA | Yes | Yes | Unclear | Unclear | Yes | Yes | Unclear | Unclear | Yes |
| Kirmani 2014 [81] | Unclear | No | Unclear | Unclear | Unclear | Yes | NA | Unclear | NA | Yes | Yes | Yes | NA | Yes | Yes | Unclear | Unclear | Yes |
| Kong 1989 [82] | Unclear | No | Yes | Unclear | No | Yes | NA | Unclear | Yes | Yes | Yes | No | NA | Yes | Yes | Unclear | Unclear | Yes |
| Kotokey 2006 [83] | Yes | No | Unclear | No | Yes | Yes | NA | Unclear | Unclear | Yes | Yes | Yes | NA | Yes | Yes | Unclear | Unclear | Yes |
| Kunz 1989 [84] | Unclear | No | Unclear | Unclear | NA | Yes | Unclear | Unclear | No | Yes | NA | No | NA | Yes | Yes | Unclear | Unclear | Yes |
| Larralde 1986 [85] | Unclear | No | Unclear | Unclear | No | No | Unclear | Unclear | No | Yes | Yes | No | NA | Yes | Yes | Unclear | Unclear | Yes |
| Lee 1993 [86] | Yes | No | Yes | Unclear | No | Yes | Unclear | Unclear | No | Yes | Yes | No | Unclear | Yes | Yes | Unclear | Unclear | Yes |
| Lee 2005a [87] | Unclear | No | Yes | Yes | No | No | Unclear | Unclear | Yes | Yes | Yes | No | Unclear | Yes | Yes | Unclear | Unclear | Yes |
| Lee 2005b [88] | Unclear | No | Yes | Yes | No | Yes | Unclear | Unclear | Yes | Yes | Yes | No | Unclear | Yes | Yes | Unclear | Unclear | Yes |
|  | Q1 | Q2 | Q3.1 | Q3.2 | Q4 | Q5 | Q6 | Q7 | Q8 | Q9.1 | Q9.2 | Q10 | Q11 | Q12 | Q13 | Q14 | Q15 | Q16 |
| Lee 2010 [89] | Unclear | No | Yes | Yes | No | Yes | Unclear | Unclear | Yes | Yes | Yes | No | Unclear | Yes | Yes | Unclear | Unclear | Yes |
| Lee 2011 [90] | Unclear | No | Yes | Yes | No | No | Unclear | Unclear | Yes | Yes | Unclear | No | NA | Yes | Yes | Unclear | Unclear | Yes |
| Lee 2019 [91] | Unclear | No | Yes | Unclear | No | Yes | NA | Unclear | Yes | Yes | Unclear | No | Unclear | Yes | Yes | Unclear | Unclear | Yes |
| Livramento 1985 [92] | Yes | No | Unclear | Unclear | NA | Yes | Unclear | Unclear | Yes | Yes | NA | No | No | No | Yes | Unclear | Unclear | Yes |
| Machado 2007 [93] | Unclear | No | Yes | Yes | No | Yes | Unclear | Unclear | Yes | Yes | Yes | Yes | NA | Yes | Yes | Unclear | Unclear | Yes |
| Machado 2013 [94] | Unclear | No | Yes | Yes | No | Yes | Unclear | Unclear | Yes | Yes | Yes | Yes | NA | Yes | Yes | Unclear | Unclear | Yes |
| Mandal 2006 [95] | Unclear | No | Yes | Yes | No | Yes | Unclear | Unclear | Yes | Yes | Yes | Yes | NA | Yes | Yes | Unclear | Unclear | Yes |
| Mandal 2008 [96] | Unclear | No | Yes | Yes | No | Yes | Unclear | Unclear | Yes | Yes | Yes | Yes | NA | Yes | Yes | Unclear | Unclear | Yes |
| Manhani 2011 [97] | Unclear | No | Yes | Yes | No | Yes | Unclear | Unclear | Yes | Yes | Yes | Unclear | Unclear | Yes | Yes | Unclear | Unclear | Yes |
| Mason 1992 [98] | Unclear | Unclear | Yes | Yes | No | Yes | Unclear | Unclear | Yes | Yes | Yes | No | Unclear | Unclear | Unclear | Unclear | Unclear | Yes |
| Mayta 2009 [99] | Unclear | No | Unclear | No | No | Yes | Yes | Yes | Unclear | Yes | Yes | Yes | NA | Yes | Yes | Unclear | Unclear | Yes |
| Minozzo 2008 [100] | Unclear | No | Unclear | Yes | Unclear | No | Unclear | Unclear | Yes | Yes | Unclear | Yes | NA | Yes | Yes | Unclear | Unclear | Yes |
| Mohammad 1984 [101] | Unclear | No | Unclear | Yes | Unclear | Yes | NA | Unclear | Yes | Yes | Yes | No | Unclear | Yes | Yes | Unclear | Unclear | Yes |
| Montenegro 1994 [102] | Unclear | No | Yes | Unclear | No | Yes | Unclear | Unclear | Yes | Yes | Yes | Yes | NA | Yes | Yes | Unclear | Unclear | Yes |
| Montero 2003 [103] | Unclear | No | Yes | Unclear | No | Yes | Unclear | Unclear | Yes | Yes | Yes | No | Unclear | Yes | Yes | Unclear | Unclear | Yes |
| Morales 2018 [104] | Unclear | No | Unclear | No | Yes | Yes | NA | Unclear | Yes | No | Yes | Yes | NA | Yes | Yes | Unclear | Unclear | Yes |
| Morillo 2020 [105] | Unclear | No | Yes | Yes | No | Yes | Unclear | Unclear | Unclear | Yes | Yes | No | NA | Yes | Yes | Unclear | Unclear | Yes |
| Nascimento 1987a [106] | Unclear | No | Yes | Unclear | No | Yes | Unclear | Unclear | Unclear | Yes | Yes | Yes | NA | Yes | Yes | Unclear | Unclear | Yes |
| Nascimento 1987b [107] | Unclear | No | Yes | No | No | Yes | Unclear | Unclear | No | Unclear | Unclear | Yes | NA | Yes | Yes | Unclear | Unclear | Yes |
| Nguekam 2003 [108] | Unclear | Yes | Unclear | Yes | Yes | Yes | NA | Yes | Yes | Yes | Yes | Yes | NA | Yes | Yes | Unclear | No | Yes |
| Nhancupe 2013 [109] | Unclear | No | Yes | Unclear | Yes | Yes | Unclear | Unclear | NA | Yes | Yes | Yes | NA | Yes | Yes | Unclear | Unclear | Yes |
| Noh 2014 [110] | Unclear | No | Yes | Yes | No | Yes | Unclear | Unclear | NA | No | No | No | Unclear | Yes | Yes | Unclear | Unclear | Yes |
| Nunes 2013 [111] | Unclear | No | Yes | Yes | No | Yes | Unclear | Unclear | Yes | Yes | Yes | Unclear | Unclear | Yes | Yes | Unclear | Unclear | Yes |
| Nunes 2017 [112] | Unclear | No | Yes | Yes | No | Yes | Unclear | Unclear | Yes | Yes | Yes | Unclear | Unclear | Yes | Yes | Unclear | Unclear | Yes |
| Oliveira 2007 [113] | Unclear | No | Yes | Yes | No | Yes | Unclear | Unclear | Yes | Yes | Yes | Yes | NA | Yes | Yes | Unclear | Unclear | Yes |
| Oliveira 2010 [114] | Unclear | No | Yes | Yes | No | Yes | Unclear | Unclear | Yes | Yes | Yes | Yes | NA | Yes | Yes | Unclear | Unclear | Yes |
| Oommen 2004 [115] | Unclear | No | Unclear | No | No | No | Unclear | Unclear | Yes | Yes | Yes | No | NA | Yes | Yes | Unclear | Unclear | Yes |
| Palacio 1998 [116] | Yes | No | Unclear | Yes | Yes | Yes | NA | Unclear | Yes | Yes | Yes | Yes | NA | Yes | Yes | Unclear | Unclear | Yes |
| Pammenter 1984 [117] | Unclear | No | Yes | Yes | No | No | Unclear | Unclear | Yes | Unclear | Yes | No | Unclear | Yes | Yes | No | Unclear | Yes |
| Pammenter 1987 [118] | Unclear | No | Yes | Yes | No | Yes | Unclear | Unclear | Yes | Yes | Yes | Yes | NA | Yes | Yes | Unclear | Unclear | Yes |
|  | Q1 | Q2 | Q3.1 | Q3.2 | Q4 | Q5 | Q6 | Q7 | Q8 | Q9.1 | Q9.2 | Q10 | Q11 | Q12 | Q13 | Q14 | Q15 | Q16 |
| Pappala 2017 [119] | Yes | No | No | Yes | No | Yes | Unclear | Unclear | Yes | Yes | No | Yes | Unclear | Yes | Yes | Unclear | Unclear | Yes |
| Parija 2004 [120] | Unclear | No | Yes | Yes | No | Yes | Yes | Yes | NA | Yes | Yes | No | NA | Yes | Yes | Unclear | Unclear | Yes |
| Parija 2006 [121] | Unclear | No | Yes | Unclear | No | Yes | Unclear | Unclear | NA | Yes | Yes | No | NA | Yes | Yes | Unclear | Unclear | Yes |
| Parkhouse 2018 [122] | Unclear | No | Unclear | Yes | NA | Yes | NA | Unclear | Yes | Yes | NA | Yes | Unclear | Yes | Yes | Unclear | Unclear | Yes |
| Parkhouse 2019 [123] | Unclear | No | Unclear | Yes | NA | Yes | NA | Unclear | NA | Yes | NA | Yes | Unclear | Yes | Yes | Unclear | Unclear | Yes |
| Peralta 2002 [124] | Unclear | No | Yes | Yes | No | No | Unclear | Unclear | Yes | Yes | Yes | Yes | NA | Yes | Yes | Unclear | Yes | Yes |
| Piña 2011 [125] | Unclear | No | Yes | Yes | No | Yes | NA | Yes | NA | Yes | Yes | No | Unclear | Yes | Yes | Unclear | Unclear | Yes |
| Plancarte 1994 [126] | Unclear | No | Yes | Yes | NA | Yes | Unclear | Unclear | Yes | Yes | NA | Yes | NA | Yes | Yes | Unclear | Unclear | Yes |
| Prabhakaran 2004 [127] | Unclear | No | Yes | Unclear | No | Yes | Yes | Yes | Yes | Yes | Yes | No | Unclear | Yes | Yes | Unclear | Unclear | Yes |
| Prabhakaran 2007 [128] | Unclear | No | Unclear | No | Yes | Yes | Yes | Yes | Yes | Yes | Yes | No | Unclear | Yes | Yes | Unclear | Unclear | Yes |
| Proano-Narvaez 2002 [129] | Unclear | No | Unclear | Yes | Unclear | Yes | Yes | Yes | Yes | Unclear | Yes | No | Unclear | Yes | Yes | Unclear | Unclear | Yes |
| Prudhivi 2015 [130] | Unclear | Yes | Unclear | Yes | NA | Yes | NA | Unclear | Yes | Yes | NA | No | NA | Yes | Unclear | Unclear | Unclear | Yes |
| Ramos-Kuri 1992 [131] | Yes | Yes | Unclear | Yes | Yes | Yes | NA | Yes | Yes | Yes | Yes | Unclear | Unclear | Yes | Yes | Unclear | Unclear | Yes |
| Ribeiro 2013 [132] | Unclear | No | Yes | Unclear | No | Yes | Unclear | Unclear | Yes | Yes | Unclear | Unclear | Unclear | Yes | Yes | Unclear | Unclear | Yes |
| Ribeiro 2014 [133] | Unclear | No | Yes | Unclear | No | Yes | Unclear | Unclear | Yes | Yes | Yes | Unclear | Unclear | Yes | Yes | Unclear | Unclear | Yes |
| Ribeiro 2019 [134] | Unclear | No | Yes | Yes | No | Yes | Unclear | Unclear | Yes | Yes | Yes | Unclear | Unclear | Yes | Yes | Unclear | Unclear | Yes |
| Rodriguez 2009 [135] | Unclear | No | Unclear | Yes | NA | No | Unclear | Unclear | Yes | Yes | NA | No | Unclear | Yes | Yes | Unclear | No | Yes |
| Rodriguez-Canul 1997 [136] | Unclear | No | Yes | Unclear | No | Yes | Unclear | Unclear | NA | Yes | Yes | No | Unclear | Yes | Yes | Unclear | Unclear | Yes |
| Rosas 1986 [137] | Unclear | No | Unclear | Yes | Yes | Yes | NA | Yes | Yes | Yes | Yes | No | Unclear | Yes | Yes | Unclear | Unclear | Yes |
| Rueda 2011 [138] | Unclear | No | Yes | No | No | Yes | NA | Yes | NA | Yes | Yes | Unclear | Unclear | Yes | Yes | Unclear | Unclear | Yes |
| Saavedra-Camacho 2021 [139] | Unclear | No | Unclear | No | NA | Yes | NA | Yes | Yes | Yes | NA | Yes | Unclear | Yes | Yes | Unclear | Unclear | Yes |
| Sadaow 2023 [140] | Unclear | No | Yes | Yes | No | Yes | NA | Unclear | NA | Yes | Yes | No | Unclear | Yes | Yes | Unclear | Unclear | Yes |
| Sahu 2009 [141] | Unclear | No | Yes | Yes | No | Yes | Unclear | Unclear | Yes | Yes | Yes | Yes | NA | Yes | Yes | Unclear | Unclear | Yes |
| Sahu 2010 [142] | Unclear | No | Yes | Unclear | No | Yes | No | Unclear | NA | Yes | Yes | Unclear | Unclear | Yes | Yes | Unclear | Unclear | Yes |
| Sahu 2014 [143] | Unclear | No | Yes | Unclear | No | Yes | Unclear | Unclear | NA | Yes | Yes | Unclear | Unclear | Yes | Yes | Unclear | Unclear | Yes |
| Sahu 2015 [144] | Unclear | Yes | Unclear | Yes | NA | Yes | NA | Unclear | NA | Yes | NA | Yes | NA | Yes | Unclear | Unclear | Unclear | Yes |
| Sako 2000 [145] | Unclear | No | Yes | Yes | No | Yes | NA | Unclear | Yes | Yes | Yes | No | Unclear | Yes | Yes | Unclear | No | Yes |
| Sako 2015 [146] | Unclear | No | Unclear | Yes | Yes | Yes | NA | Yes | Yes | Yes | Yes | Yes | NA | Yes | Yes | Unclear | Unclear | Yes |
| Salazar-Anton 2011 [147] | Unclear | No | Unclear | Unclear | No | Yes | NA | Unclear | NA | Yes | Yes | Yes | NA | Yes | Yes | Unclear | Unclear | Yes |
| Sanchez 1999a [148] | Yes | Yes | Unclear | Yes | Yes | Yes | NA | Unclear | Yes | Yes | Yes | Yes | NA | Yes | Yes | Unclear | Unclear | Yes |
|  | Q1 | Q2 | Q3.1 | Q3.2 | Q4 | Q5 | Q6 | Q7 | Q8 | Q9.1 | Q9.2 | Q10 | Q11 | Q12 | Q13 | Q14 | Q15 | Q16 |
| Sanchez 1999b [149] | Yes | Yes | Unclear | Yes | NA | Yes | NA | Yes | NA | Yes | NA | Yes | NA | Yes | Yes | Unclear | Unclear | Yes |
| Schantz 1994 [150] | Yes | Yes | Yes | Yes | NA | Yes | Unclear | Yes | Yes | Yes | NA | Yes | NA | Yes | Unclear | Unclear | Unclear | Yes |
| Scheel 2005 [151] | Unclear | No | Yes | Yes | No | No | Unclear | Unclear | Yes | Yes | Yes | No | NA | Yes | Yes | Unclear | Unclear | Yes |
| Segamwenge 2016 [152] | Unclear | Yes | Unclear | Yes | Yes | Yes | NA | Unclear | Unclear | Yes | Yes | Yes | NA | Yes | Unclear | Unclear | Unclear | Yes |
| Shiguekawa 2000 [153] | Unclear | No | Yes | Unclear | No | No | Unclear | Unclear | Yes | Yes | No | Yes | NA | Yes | Yes | Unclear | Unclear | Yes |
| Shukla 2008 [154] | Unclear | No | Yes | Yes | Yes | Yes | Yes | Yes | NA | Yes | Yes | No | NA | Yes | Yes | Unclear | Unclear | Yes |
| Stelzle 2024 [155] | Yes | Yes | Unclear | Yes | Yes | No | Unclear | Yes | NA | Unclear | Unclear | Yes | NA | Yes | Yes | Yes | No | Yes |
| Suzuki 2011 [156] | Unclear | No | Yes | Unclear | No | Yes | Unclear | Unclear | Yes | Yes | Yes | Unclear | Unclear | Yes | Yes | Unclear | Unclear | Yes |
| Téllez Girón 1984 [157] | Unclear | No | Yes | Unclear | Yes | Yes | Unclear | Unclear | Yes | Yes | Yes | Yes | Unclear | Yes | Yes | Unclear | Unclear | Yes |
| Toribio 2023a [158] | Yes | No | Unclear | No | No | Yes | NA | Yes | NA | Yes | Yes | Unclear | Unclear | Unclear | Yes | Unclear | Unclear | Yes |
| Toribio 2023b [159] | Unclear | No | Unclear | Yes | No | Yes | Yes | Yes | NA | Yes | Yes | No | Unclear | Yes | Yes | Unclear | Unclear | Yes |
| Tsang 1989 [160] | Unclear | No | Yes | Unclear | No | Yes | NA | Unclear | Yes | Yes | Yes | Yes | NA | Yes | Yes | Unclear | Unclear | Yes |
| Vasudevan 2022 [161] | Unclear | No | Unclear | No | NA | Yes | NA | Yes | Yes | Yes | NA | No | Unclear | Yes | Yes | Unclear | Yes | Yes |
| Verastegui 2003 [162] | Unclear | No | Yes | No | No | Yes | No | Unclear | NA | Yes | Yes | Yes | NA | Yes | Yes | Unclear | Unclear | Yes |
| Wang 2004 [163] | Unclear | No | Yes | Yes | Unclear | Yes | NA | Unclear | Yes | Yes | Yes | Yes | NA | Yes | Yes | Unclear | No | Yes |
| Wilson 1991 [164] | Unclear | No | Unclear | Yes | NA | Yes | NA | Unclear | NA | Yes | NA | No | Unclear | Yes | Yes | Unclear | Unclear | Yes |
| Yang 1998 [165] | Yes | No | Yes | Unclear | No | Yes | NA | Unclear | NA | Yes | No | No | NA | Yes | Yes | Unclear | Unclear | Yes |
| Zammarchi 2018 [166] | Yes | Yes | Yes | Yes | NA | No | Unclear | Yes | Yes | Yes | NA | Yes | NA | Yes | Unclear | Unclear | Unclear | Yes |
| Zea-Vera 2013 [167] | Yes | No | Unclear | No | NA | Yes | Unclear | Unclear | No | Yes | NA | Yes | Yes | Yes | Yes | Unclear | Yes | Yes |
| Zimic 2009 [168] | Unclear | No | Yes | No | No | No | Unclear | Unclear | No | Yes | Yes | No | Unclear | Yes | Yes | Unclear | Unclear | Yes |
| Zulu 2024 [169] | Yes | Yes | Unclear | Yes | Yes | No | Yes | Yes | NA | Yes | Yes | Yes | NA | Yes | Yes | Yes | No | Yes |

**References**

1. Agrawal I, Mittal M, Mittal G. A comparative study of single versus multiple cysts of neurocysticercosis based on their clinico-serological profile. Journal of Clinical and Diagnostic Research. 2017;11(11):OC25-OC8.

2. Agudelo P, Botero D, Palacio LG. [Evaluation of the ELISA method for diagnosis of human cysticercosis in an endemic region]. Biomedica. 2005;25(4):488-95.

3. Aguilar-Rebolledo F, Meza-Lucas A, Torres J, Cedillo-Rivera R, Enciso A, Garcia RC, et al. Evaluation of the enzyme-linked immunoelectrotransfer blot assay for diagnosis of neurocysticercosis in children. J Child Neurol. 2002;17(6):416-20.

4. Ahn CS, Bae YA, Kim SH, Kim JG, Yu JR, Yang HJ, et al. Spatiotemporal Expression Patterns and Antibody Reactivity of Taeniidae Endophilin B1. Journal of clinical microbiology. 2016;54(10):2553-62.

5. Anadure RK, Wilson V, Ragini, Saxena R, Mohimen A, Sivasankar R, et al. A Multicentric Study on the Clinical Profile, Serology, Neuroimaging and Treatment of Neurocysticercosis. Journal of Clinical and Diagnostic Research. 2020;14(10):OC10-OC4.

6. Arora N, Kaur R, Rawat SS, Kumar A, Singh AK, Tripathi S, et al. Evaluation of Taenia solium cyst fluid-based enzyme linked immunoelectro transfer blot for Neurocysticercosis diagnosis in urban and highly endemic rural population of North India. Clinica chimica acta; international journal of clinical chemistry. 2020;508:16-21.

7. Arruda GC, da Silva AD, Quagliato EM, Maretti MA, Rossi CL. Evaluation of Taenia solium and Taenia crassiceps cysticercal antigens for the serodiagnosis of neurocysticercosis. Tropical medicine & international health : TM & IH. 2005;10(10):1005-12.

8. Arruda GC, Quagliato EM, Rossi CL. Intrathecal synthesis of specific immunoglobulin G antibodies in neurocysticercosis: evaluation of antibody concentrations by enzyme-linked immunosorbent assay using a whole cysticercal extract and cyst vesicular fluid as antigens. Diagn Microbiol Infect Dis. 2006;54(1):45-9.

9. Arthi E, Selvi R. Seroprevalence of Neurocysticercosis among Epilepsy Patients in Chennai, Southern India- A Cross-sectional Study. Journal of Clinical and Diagnostic Research. 2021;15(12):DC01-DC4.

10. Astudillo OG, Diego RG, Irazu L, Rodriguez M. Evaluation of the ELISA method for the detection of anti-cysticercus antibodies in human sera. Acta Bioquimica Clinica Latinoamericana. 2019;53(3):353-60.

11. Atluri SR, Singhi P, Khandelwal N, Malla N. Evaluation of excretory secretory and 10-30 kDa antigens of Taenia solium Cysticerci by EITB assay for the diagnosis of neurocysticercosis. Parasite Immunol. 2009;31(3):151-5.

12. Atluri SR, Singhi P, Khandelwal N, Malla N. Neurocysticercosis immunodiagnosis using Taenia solium cysticerci crude soluble extract, excretory secretory and lower molecular mass antigens in serum and urine samples of Indian children. Acta Trop. 2009;110(1):22-7.

13. Atluri VS, Singhi PD, Khandelwal N, Malla N. 2D-PAGE analysis of Taenia solium metacestode 10-30 kDa antigens for the serodiagnosis of neurocysticercosis in children. Acta Trop. 2011;118(2):165-9.

14. Ayala-Sulca E, Miranda-Ulloa E. [Evaluation of enzyme-linked immunoelectrotransfer blot test using purified native antigen mix from cisticercus fluid of Taenia solium for diagnosis of human cysticercosis]. Revista peruana de medicina experimental y salud publica. 2015;32(3):485-91.

15. Bae YA, Jeong YT, Chung JY, Kim SH, Mahanta J, Feng Z, et al. A recombinant chimeric antigen toward a standardized serodiagnosis of Taenia solium neurocysticercosis. Proteomics Clin Appl. 2008;2(12):1596-610.

16. Bae YA, Yeom JS, Wang H, Kim SH, Ahn CS, Kim JT, et al. Taenia solium metacestode fasciclin-like protein is reactive with sera of chronic neurocysticercosis. Tropical medicine & international health : TM & IH. 2014;19(6):719-25.

17. Baily GG, Mason PR, Trijssenar FE, Lyons NF. Serological diagnosis of neurocysticercosis: evaluation of ELISA tests using cyst fluid and other components of Taenia solium cysticerci as antigens. Transactions of the Royal Society of Tropical Medicine and Hygiene. 1988;82(2):295-9.

18. Barcelos IS, Ferreira MS, Moura LP, Biondi GF, Costa-Cruz JM. Use of the paired samples (cerebrospinal fluid and serum) in immunodiagnostic of active and inactive human neurocysticercosis. Mem Inst Oswaldo Cruz. 2005;100(4):427-9.

19. Barcelos IS, Moura LP, Costa VP, Ferreira MS, Costa-Cruz JM. Taenia solium metacestode immunodominant peptides recognized by IgG antibodies in cerebrospinal fluid and serum paired samples from patients with active and inactive neurocysticercosis. Mem Inst Oswaldo Cruz. 2007;102(6):713-7.

20. Biswas R, Parija SC, Narayan SK. Dot-ELISA for the diagnosis of neurocysticercosis. Revista do Instituto de Medicina Tropical de Sao Paulo. 2004;46(5):249-52.

21. Brizzi K, Pelden S, Tshokey T, Nirola DK, Diamond MB, Klein JP, et al. Neurocysticercosis in Bhutan: a cross-sectional study in people with epilepsy. Trans R Soc Trop Med Hyg. 2016;110(9):517-26.

22. Bueno EC, Vaz AJ, Machado LD, Livramento JA, Mielle SR. Specific Taenia crassiceps and Taenia solium antigenic peptides for neurocysticercosis immunodiagnosis using serum samples. Journal of clinical microbiology. 2000;38(1):146-51.

23. Bueno EC, Vaz AJ, Machado LD, Livramento JA. Neurocysticercosis: detection of IgG, IgA and IgE antibodies in cerebrospinal fluid, serum and saliva samples by ELISA with Taenia solium and Taenia crassiceps antigens. Arq Neuropsiquiatr. 2000;58(1):18-24.

24. Bueno EC, Scheel CM, Vaz AJ, Machado LR, Livramento JA, Takayanagui OM, et al. Application of synthetic 8-kD and recombinant GP50 antigens in the diagnosis of neurocysticercosis by enzyme-linked immunosorbent assay. The American journal of tropical medicine and hygiene. 2005;72(3):278-83.

25. Carod JF, Randrianarison M, Razafimahefa J, Ramahefarisoa RM, Rakotondrazaka M, Debruyne M, et al. Evaluation of the performance of 5 commercialized enzyme immunoassays for the detection of Taenia solium antibodies and for the diagnosis of neurocysticercosis. Diagnostic microbiology and infectious disease. 2012;72(1):85-9.

26. Carrara GMP, Silva GB, Faria LS, Nunes DS, Ribeiro VS, Lopes CA, et al. IgY antibody and human neurocysticercosis: a novel approach on immunodiagnosis using Taenia crassiceps hydrophobic antigens. Parasitology. 2020;147(2):240-7.

27. Castillo Y, Rodriguez S, García HH, Brandt J, Van Hul A, Silva M, et al. Urine antigen detection for the diagnosis of human neurocysticercosis. Am J Trop Med Hyg. 2009;80(3):379-83.

28. Castillo Y, Toribio LM, Guzman C, Arroyo G, Espinoza C, Saavedra H, et al. Consistent Measurement of Parasite-Specific Antigen Levels in Sera of Patients with Neurocysticercosis Using Two Different Monoclonal Antibody (mAb)-Based Enzyme-Linked Immunosorbent Assays. Pathogens. 2023;12(4).

29. Chang KH, Kim WS, Cho SY, Han MC, Kim CW. Comparative evaluation of brain CT and ELISA in the diagnosis of neurocysticercosis. AJNR Am J Neuroradiol. 1988;9(1):125-30.

30. Chuang C, Xing-Wang C, Jian-Zhong D, Tiao-Ying L. [Evaluation of ELISA kit for detection of serum specific IgG antibodies against Taenia solium in diagnosis of human cysticercosis]. Zhongguo xue xi chong bing fang zhi za zhi = Chinese journal of schistosomiasis control. 2017;29(2):228-30.

31. Cho SY, Kim SI, Kang SY, Choi DY, Suk JS, Choi KS, et al. Evaluation of enzyme-linked immunosorbent assay in serological diagnosis of human neurocysticercosis using paired samples of serum and cerebrospinal fluid. Kisaengchunghak Chapchi. 1986;24(1):25-41.

32. Chung JY, Bahk YY, Huh S, Kang SY, Kong Y, Cho SY. A recombinant 10-kDa protein of Taenia solium metacestodes specific to active neurocysticercosis. The Journal of infectious diseases. 1999;180(4):1307-15.

33. Corona T, Pascoe D, González-Barranco D, Abad P, Landa L, Estañol B. Anticysticercous antibodies in serum and cerebrospinal fluid in patients with cerebral cysticercosis. J Neurol Neurosurg Psychiatry. 1986;49(9):1044-9.

34. Corstjens PL, de Dood CJ, Priest JW, Tanke HJ, Handali S. Feasibility of a lateral flow test for neurocysticercosis using novel up-converting nanomaterials and a lightweight strip analyzer. PLoS neglected tropical diseases. 2014;8(7):e2944.

35. da Silva MR, Maia AA, Espíndola NM, Machado Ldos R, Vaz AJ, Henrique-Silva F. Recombinant expression of Taenia solium TS14 antigen and its utilization for immunodiagnosis of neurocysticercosis. Acta Trop. 2006;100(3):192-8.

36. da Silva GB, Faria LSD, Lopes CA, Nunes DS, Ribeiro VS, de Sousa JEN, et al. Egg yolk immunoglobulin Y as a promising tool to detect immune complexes in neurocysticercosis serum samples. Transactions of the Royal Society of Tropical Medicine and Hygiene. 2020;114(8):585-92.

37. da Silva RV, Manhani MN, Cardoso R, Vieira CU, Goulart LR, Costa-Cruz JM. Selection of high affinity peptide ligands for detection of circulating antibodies in neurocysticercosis. Immunology letters. 2010;129(2):94-9.

38. da Silva RV, Manhani MN, Costa-Cruz JM. IgA detection in human neurocysticercosis using different preparations of heterologous antigen. Parasitology research. 2010;107(1):221-5.

39. Davelois K, Escalante H, Jara C. [Western Blot diagnostic yield for simultaneous antibody-detection in patients with human cysticercosis, hydatidosis, and human fascioliasis]. Rev Peru Med Exp Salud Publica. 2016;33(4):616-24.

40. De Macedo HW, Peralta RHS, Cipriano A, Sarmento MR, Vaz AJ, Peralta JM. Evaluation of immunological tests for the diagnosis of neurocysticercosis. Jornal Brasileiro de Patologia e Medicina Laboratorial. 2002;38(2):93-103.

41. Dermauw V, Carabin H, Cissé A, Millogo A, Tarnagda Z, Ganaba R, et al. Evaluating the Recombinant T24H Enzyme-Linked Immunoelectrotransfer Blot Assay for the Diagnosis of Neurocysticercosis in a Panel of Samples from a Large Community-Based Randomized Control Trial in 60 Villages in Burkina Faso. The American journal of tropical medicine and hygiene. 2018;98(2):565-9.

42. Diaz JF, Verastegui M, Gilman RH, Tsang VC, Pilcher JB, Gallo C, et al. Immunodiagnosis of human cysticercosis (Taenia solium): a field comparison of an antibody-enzyme-linked immunosorbent assay (ELISA), an antigen-ELISA, and an enzyme-linked immunoelectrotransfer blot (EITB) assay in Peru. The Cysticercosis Working Group in Peru (CWG). The American journal of tropical medicine and hygiene. 1992;46(5):610-5.

43. Eom KS, Cho SY, Rim HJ. Comparative evaluation of indirect immunofluorescent antibody test with enzyme-linked immunosorbent assay in serodiagnosis of human neurocysticercosis. Kisaengch'unghak chapchi The Korean journal of parasitology. 1988;26(1):27-32.

44. Espinoza B, Ruiz-Palacios G, Tovar A, Sandoval MA, Plancarte A, Flisser A. Characterization by enzyme-linked immunosorbent assay of the humoral immune response in patients with neurocysticercosis and its application in immunodiagnosis. Journal of clinical microbiology. 1986;24(4):536-41.

45. Espíndola NM, Iha AH, Fernandes I, Takayanagui OM, Machado Ldos R, Livramento JA, et al. Cysticercosis immunodiagnosis using 18- and 14-kilodalton proteins from Taenia crassiceps cysticercus antigens obtained by immunoaffinity chromatography. Journal of clinical microbiology. 2005;43(7):3178-84.

46. Feldman M, Plancarte A, Sandoval M, Wilson M, Flisser A. Comparison of two assays (EIA and EITB) and two samples (saliva and serum) for the diagnosis of neurocysticercosis. Transactions of the Royal Society of Tropical Medicine and Hygiene. 1990;84(4):559-62.

47. Ferrer E, Cortez MM, Perez H, De la Rosa M, de Noya BA, D'Avila I, et al. Serological evidence for recent exposure to Taenia solium in Venezuelan Amerindians. Am J Trop Med Hyg. 2002;66(2):170-4.

48. Ferrer E, Moyano E, Benitez L, González LM, Bryce D, Foster-Cuevas M, et al. Cloning and characterization of Taenia saginata paramyosin cDNA. Parasitol Res. 2003;91(1):60-7.

49. Ferrer E, González LM, Foster-Cuevas M, Cortéz MM, Dávila I, Rodríguez M, et al. Taenia solium: characterization of a small heat shock protein (Tsol-sHSP35.6) and its possible relevance to the diagnosis and pathogenesis of neurocysticercosis. Exp Parasitol. 2005;110(1):1-11.

50. Ferrer E, Cortéz MM, Cabrera Z, Rojas G, Dávila I, Alarcón de Noya B, et al. Oncospheral peptide-based ELISAs as potential seroepidemiological tools for Taenia solium cysticercosis/neurocysticercosis in Venezuela. Transactions of the Royal Society of Tropical Medicine and Hygiene. 2005;99(8):568-76.

51. Ferrer E, Bonay P, Foster-Cuevas M, González LM, Dávila I, Cortéz MM, et al. Molecular cloning and characterisation of Ts8B1, Ts8B2 and Ts8B3, three new members of the Taenia solium metacestode 8 kDa diagnostic antigen family. Mol Biochem Parasitol. 2007;152(1):90-100.

52. Ferrer E, González LM, Martínez-Escribano JA, González-Barderas ME, Cortéz MM, Dávila I, et al. Evaluation of recombinant HP6-Tsag, an 18 kDa Taenia saginata oncospheral adhesion protein, for the diagnosis of cysticercosis. Parasitol Res. 2007;101(3):517-25.

53. Ferrer E, Martínez-Escribano JA, Barderas ME, González LM, Cortéz MM, Dávila I, et al. Peptide epitopes of the Taenia solium antigen Ts8B2 are immunodominant in human and porcine cysticercosis. Mol Biochem Parasitol. 2009;168(2):168-71.

54. Ferrer E, Sánchez J, Milano A, Alvarez S, La Rosa R, Lares M, et al. Diagnostic epitope variability within Taenia solium 8 kDa antigen family: implications for cysticercosis immunodetection. Exp Parasitol. 2012;130(1):78-85.

55. Fleury A, Bouteille B, Garcia E, Marquez C, Preux PM, Escobedo F, et al. Neurocysticercosis: validity of ELISA after storage of whole blood and cerebrospinal fluid on paper. Trop Med Int Health. 2001;6(9):688-93.

56. Fleury A, Hernández M, Avila M, Cárdenas G, Bobes RJ, Huerta M, et al. Detection of HP10 antigen in serum for diagnosis and follow-up of subarachnoidal and intraventricular human neurocysticercosis. J Neurol Neurosurg Psychiatry. 2007;78(9):970-4.

57. Flisser A, Woodhouse E, Larralde C. Human cysticercosis: antigens, antibodies and non-responders. Clin Exp Immunol. 1980;39(1):27-37.

58. Foyaca-Sibat H, Cowan LD, Carabin H, Targonska I, Anwary MA, Serrano-Ocaña G, et al. Accuracy of serological testing for the diagnosis of prevalent neurocysticercosis in outpatients with epilepsy, Eastern Cape Province, South Africa. PLoS neglected tropical diseases. 2009;3(12):e562.

59. Gabriël S, Blocher J, Dorny P, Abatih EN, Schmutzhard E, Ombay M, et al. Added value of antigen ELISA in the diagnosis of neurocysticercosis in resource poor settings. PLoS neglected tropical diseases. 2012;6(10):e1851.

60. Garcia HH, Parkhouse RM, Gilman RH, Montenegro T, Bernal T, Martinez SM, et al. Serum antigen detection in the diagnosis, treatment, and follow-up of neurocysticercosis patients. Transactions of the Royal Society of Tropical Medicine and Hygiene. 2000;94(6):673-6.

61. Garcia HH, Gonzalez AE, Gilman RH, Bernal T, Rodriguez S, Pretell EJ, et al. Circulating parasite antigen in patients with hydrocephalus secondary to neurocysticercosis. Am J Trop Med Hyg. 2002;66(4):427-30.

62. Garcia HH, O'Neal SE, Noh J, Handali S. Laboratory Diagnosis of Neurocysticercosis (Taenia solium). J Clin Microbiol. 2018;56(9).

63. Gekeler F, Eichenlaub S, Mendoza EG, Sotelo J, Hoelscher M, Löscher T. Sensitivity and specificity of ELISA and immunoblot for diagnosing neurocysticercosis. European journal of clinical microbiology & infectious diseases : official publication of the European Society of Clinical Microbiology. 2002;21(3):227-9.

64. Gonçalves Fde A, Machado GA, Oliveira HB, Rezende MT, Mineo JR, Costa-Cruz JM. Hydrophobic fraction of Taenia saginata metacestodes, rather than hydrophilic fraction, contains immunodominant markers for diagnosing human neurocysticercosis. Revista da Sociedade Brasileira de Medicina Tropical. 2010;43(3):254-9.

65. González E, Robles Y, Govezensky T, Bobes RJ, Gevorkian G, Manoutcharian K. Isolation of neurocysticercosis-related antigens from a genomic phage display library of Taenia solium. J Biomol Screen. 2010;15(10):1268-73.

66. Greene RM, Wilkins PP, Tsang VC. Diagnostic glycoproteins of Taenia solium cysts share homologous 14- and 18-kDa subunits. Mol Biochem Parasitol. 1999;99(2):257-61.

67. Hancock K, Pattabhi S, Whitfield FW, Yushak ML, Lane WS, Garciac HH, et al. Characterization and cloning of T24, a Taenia solium antigen diagnostic for cysticercosis. Molecular and Biochemical Parasitology. 2006;147(1):109-17.

68. Handali S, Klarman M, Gaspard AN, Noh J, Lee YM, Rodriguez S, et al. Multiantigen print immunoassay for comparison of diagnostic antigens for Taenia solium cysticercosis and taeniasis. Clinical and vaccine immunology : CVI. 2010;17(1):68-72.

69. Handali S, Klarman M, Gaspard AN, Dong XF, LaBorde R, Noh J, et al. Development and Evaluation of a Magnetic Immunochromatographic Test To Detect Taenia solium, Which Causes Taeniasis and Neurocysticercosis in Humans. Clinical and vaccine immunology. 2010;17(4):631-7.

70. Hell RC, Amim P, de Andrade HM, de Avila RA, Felicori L, Oliveira AG, et al. Immunodiagnosis of human neurocysticercosis using a synthetic peptide selected by phage-display. Clinical immunology (Orlando, Fla). 2009;131(1):129-38.

71. Hernández M, Astudillo OG, Diego G, de-la-Rosa-Arana JL, Meza-Lucas A, García-Rodea R, et al. Immunodiagnosis of human neurocysticercosis: comparative performance of serum diagnostic tests in Mexico. Parasitol Res. 2019;118(10):2891-9.

72. Hernández-González A, Noh J, Perteguer MJ, Gárate T, Handali S. Comparison of T24H-his, GST-T24H and GST-Ts8B2 recombinant antigens in western blot, ELISA and multiplex bead-based assay for diagnosis of neurocysticercosis. Parasites & vectors. 2017;10(1):237.

73. Hernández-González A, González-Bertolín B, Urrea L, Fleury A, Ferrer E, Siles-Lucas M, et al. Multiple-bead assay for the differential serodiagnosis of neglected human cestodiases: Neurocysticercosis and cystic echinococcosis. PLoS neglected tropical diseases. 2022;16(1):e0010109.

74. Hubert K, Andriantsimahavandy A, Michault A, Frosch M, Mühlschlegel FA. Serological diagnosis of human cysticercosis by use of recombinant antigens from Taenia solium cysticerci. Clinical and diagnostic laboratory immunology. 1999;6(4):479-82.

75. Husain N, Jyotsna, Bagchi M, Husain M, Mishra MK, Gupta S. Evaluation of cysticercus fasciolaris antigen for the immunodiagnosis of neurocysticercosis. Neurology India. 2001;49(4):375-9.

76. Intapan PM, Khotsri P, Kanpittaya J, Chotmongkol V, Maleewong W, Morakote N. Evaluation of IgG4 and total IgG antibodies against cysticerci and peptide antigens for the diagnosis of human neurocysticercosis by ELISA. Asian Pacific journal of allergy and immunology. 2008;26(4):237-44.

77. Ishida MM, Rubinsky-Elefant G, Ferreira AW, Hoshino-Shimizu S, Vaz AJ. Helminth antigens (Taenia solium, Taenia crassiceps, Toxocara canis, Schistosoma mansoni and Echinococcus granulosus) and cross-reactivities in human infections and immunized animals. Acta tropica. 2003;89(1):73-84.

78. Ishida MM, Peralta RH, Livramento JA, Hoshino-Shimizu S, Peralta JM, Vaz AJ. Serodiagnosis of neurocysticercosis in patients with epileptic seizure using ELISA and immunoblot assay. Revista do Instituto de Medicina Tropical de Sao Paulo. 2006;48(6):343-6.

79. Iudici Neto F, Pianetti-Filho G, Araújo RN, Nascimento E. Immunodiagnosis of human neurocysticercosis by using semi-purified scolex antigens from Taenia solium cysticerci. Revista da Sociedade Brasileira de Medicina Tropical. 2007;40(2):163-9.

80. Jiménez L, Castro-Nolasco NK, Fleury A, Díaz-Camacho SP, Ochoa-Sánchez A, Landa A. Evaluation of recombinant glutathione transferase 26 kDa, thioredoxin-1, and endophilin B1 of Taenia solium in the diagnosis of human neurocysticercosis. Acta tropica. 2022;227:106294.

81. Kirmani S KH, Khalid M, Urfi. Sensitivity of IgG ELISA for diagnosing neurocysticercosis in a tertiary care hospital of North India. International Journal of Current Microbiology and Applied Sciences. 2014;3(8):673-9.

82. Kong Y, Kang SY, Cho SY, Min DY. Cross-reacting and specific antigenic components in cystic fluid from metacestodes of Echinococcus granulosus and Taenia solium. Kisaengchunghak Chapchi. 1989;27(2):131-9.

83. Kotokey RK, Lynrah KG, De A. A clinico-serological study of neurocysticercosis in patients with ring enhancing lesions in CT scan of brain. The Journal of the Association of Physicians of India. 2006;54:366-70.

84. Kunz J, Kalinna B, Watschke V, Geyer E. Taenia crassiceps metacestode vesicular fluid antigens shared with the Taenia solium larval stage and reactive with serum antibodies from patients with neurocysticercosis. Zentralblatt fur Bakteriologie : international journal of medical microbiology. 1989;271(4):510-20.

85. Larralde C, Laclette JP, Owen CS, Madrazo I, Sandoval M, Bojalil R, et al. Reliable serology of Taenia solium cysticercosis with antigens from cyst vesicular fluid: ELISA and hemagglutination tests. The American journal of tropical medicine and hygiene. 1986;35(5):965-73.

86. Lee JH, Kong Y, Ryu JY, Cho SY. Applicability of ABC-ELISA and protein A-ELISA in serological diagnosis of cysticercosis. The Korean journal of parasitology. 1993;31(1):49-56.

87. Lee EG, Bae YA, Jeong YT, Chung JY, Je EY, Kim SH, et al. Proteomic analysis of a 120 kDa protein complex in cyst fluid of Taenia solium metacestode and preliminary evaluation of its value for the serodiagnosis of neurocysticercosis. Parasitology. 2005;131(Pt 6):867-79.

88. Lee EG, Lee MY, Chung JY, Je EY, Bae YA, Na BK, et al. Feasibility of baculovirus-expressed recombinant 10-kDa antigen in the serodiagnosis of Taenia solium neurocysticercosis. Transactions of the Royal Society of Tropical Medicine and Hygiene. 2005;99(12):919-26.

89. Lee EG, Bae YA, Kim SH, Díaz-Camacho SP, Nawa Y, Kong Y. Serodiagnostic reliability of single-step enriched low-molecular weight proteins of Taenia solium metacestode of American and Asian isolates. Transactions of the Royal Society of Tropical Medicine and Hygiene. 2010;104(10):676-83.

90. Lee YM, Handali S, Hancock K, Pattabhi S, Kovalenko VA, Levin A, et al. Serologic Diagnosis of Human Taenia solium Cysticercosis by Using Recombinant and Synthetic Antigens in QuickELISA (TM). American journal of tropical medicine and hygiene. 2011;84(4):587-93.

91. Lee C, Noh J, O'Neal SE, Gonzalez AE, Garcia HH, Handali S. Feasibility of a point-of-care test based on quantum dots with a mobile phone reader for detection of antibody responses. PLoS Negl Trop Dis. 2019;13(10):e0007746.

92. Livramento JA, Costa JM, Machado LR, Nóbrega JP, Spina-França A. [ELISA (IgG and IgM) of the CSF and serum in neurocysticercosis under treatment with praziquantel: comparison with complement fixation reactions and immunofluorescence]. Arquivos de neuro-psiquiatria. 1985;43(3):267-74.

93. Machado GA, Santiago FM, Mineo JR, Costa-Cruz JM. Assessment of antigenic fractions of varying hydrophobicity from Taenia solium metacestodes for the diagnosis of human neurocysticercosis. Tropical medicine & international health : TM & IH. 2007;12(11):1369-76.

94. Machado GA, Oliveira HB, Gennari-Cardoso ML, Mineo JR, Costa-Cruz JM. Serodiagnosis of human neurocysticercosis using antigenic components of Taenia solium metacestodes derived from the unbound fraction from jacalin affinity chromatography. Memorias do Instituto Oswaldo Cruz. 2013;108(3):368-75.

95. Mandal J, Singhi PD, Khandelwal N, Malla N. Evaluation of ELISA and dot blots for the serodiagnosis of neurocysticercosis, in children found to have single or multiple enhancing lesions in computerized tomographic scans of the brain. Ann Trop Med Parasitol. 2006;100(1):39-48.

96. Mandal J, Singhi PD, Khandelwal N, Malla N. Evaluation of lower molecular mass (20-24 kDa) Taenia solium cysticercus antigen fraction by ELISA and dot blot for the serodiagnosis of neurocysticercosis in children. Parasitol Res. 2008;102(5):1097-101.

97. Manhani MN, Ribeiro VS, Cardoso R, Ueira-Vieira C, Goulart LR, Costa-Cruz JM. Specific phage-displayed peptides discriminate different forms of neurocysticercosis by antibody detection in the serum samples. Parasite immunology. 2011;33(6):322-9.

98. Mason P, Houston S, Gwanzura L. Neurocysticercosis: experience with diagnosis by ELISA serology and computerised tomography in Zimbabwe. The Central African journal of medicine. 1992;38(4):149-54.

99. Mayta H, Hancock K, Gilman RH, Zamudio R, Castillo JP, Levine MZ, et al. A novel Taenia solium protein that resembles troponin T proteins. The Journal of parasitology. 2009;95(3):591-7.

100. Minozzo JC, De Moura J, Almeida SM, Thomaz-Soccol V. Crude antigen from Taenia crassiceps cysticercus used as heterologous antigen in ELISA and in EITB for neurocysticercosis diagnosis of patients from Paraná-Brazil. Brazilian Archives of Biology and Technology. 2008;51(6):1127-37.

101. Mohammad IN, Heiner DC, Miller BL, Goldberg MA, Kagan IG. Enzyme-linked immunosorbent assay for the diagnosis of cerebral cysticercosis. J Clin Microbiol. 1984;20(4):775-9.

102. Montenegro T, Gilman RH, Castillo R, Tsang V, Brandt J, Guevara A, et al. The diagnostic importance of species specific and cross-reactive components of Taenia solium, Echinococcus granulosus, and Hymenolepis nana. Rev Inst Med Trop Sao Paulo. 1994;36(4):327-34.

103. Montero E, González LM, Harrison LJ, Parkhouse RM, Gárate T. Taenia solium cDNA sequence encoding a putative immunodiagnostic antigen for human cysticercosis. Journal of chromatography B, Analytical technologies in the biomedical and life sciences. 2003;786(1):255-69.

104. Morales J, Martínez JJ, Villalobos N, Hernández M, Ramírez R, Salgado-Estrada B, et al. Persistent Taenia solium Cysticercosis In the State of Morelos, Mexico: Human and Porcine Seroprevalence. The Journal of parasitology. 2018;104(5):465-72.

105. Morillo M, Noguera C, Gallego L, Fernández Z, Mata M, Khattar S, et al. Characterization and evaluation of three new recombinant antigens of Taenia solium for the immunodiagnosis of cysticercosis. Molecular and biochemical parasitology. 2020;240:111321.

106. Nascimento E, Nogueira PM, Tavares CA. Improved immunodiagnosis of human cysticercosis with scolex protein antigens. Parasitology research. 1987;73(5):446-50.

107. Nascimento E, Tavares CA, Lopes JD. Immunodiagnosis of human cysticercosis (Taenia solium) with antigens purified by monoclonal antibodies. Journal of clinical microbiology. 1987;25(7):1181-5.

108. Nguekam, Zoli AP, Ongolo-Zogo P, Dorny P, Brandt J, Geerts S. Follow-up of neurocysticercosis patients after treatment using an antigen detection ELISA. Parasite. 2003;10(1):65-8.

109. Nhancupe N, Salazar-Anton F, Noormahomed EV, Afonso S, Lindh J. Further characterization of Tsol-p27 as a diagnostic antigen in sub-Saharan Africa. Experimental parasitology. 2013;135(3):573-9.

110. Noh J, Rodriguez S, Lee YM, Handali S, Gonzalez AE, Gilman RH, et al. Recombinant protein- and synthetic peptide-based immunoblot test for diagnosis of neurocysticercosis. Journal of clinical microbiology. 2014;52(5):1429-34.

111. Nunes Dda S, Gonzaga HT, Ribeiro Vda S, da Cunha JP, Jr., Costa-Cruz JM. Taenia saginata metacestode antigenic fractions obtained by ion-exchange chromatography: potential source of immunodominant markers applicable in the immunodiagnosis of human neurocysticercosis. Diagnostic microbiology and infectious disease. 2013;76(1):36-41.

112. Nunes DS, Gonzaga HT, Ribeiro VS, Cunha-Júnior JP, Costa-Cruz JM. Usefulness of gel filtration fraction as potential biomarker for neurocysticercosis in serum: towards a new diagnostic tool. Parasitology. 2017;144(4):426-35.

113. Oliveira HB, Machado GA, Cabral DD, Costa-Cruz JM. Application of Taenia saginata metacestodes as an alternative antigen for the serological diagnosis of human neurocysticercosis. Parasitology research. 2007;101(4):1007-13.

114. Oliveira HB, Machado GA, Mineo JR, Costa-Cruz JM. Taenia saginata metacestode antigenic fractions without affinity to concanavalin A are an important source of specific antigens for the diagnosis of human neurocysticercosis. Clinical and vaccine immunology : CVI. 2010;17(4):638-44.

115. Oommen A, Prabhakaran V, Rajshekhar V, Murrell KD. Clinical immunodiagnosis of neurocysticercosis: the single cyst challenge. Southeast Asian Journal of Tropical Medicine and Public Health. 2004;35:227-30.

116. Palacio LG, Jiménez I, Garcia HH, Jiménez ME, Sánchez JL, Noh J, et al. Neurocysticercosis in persons with epilepsy in Medellín, Colombia. The Neuroepidemiological Research Group of Antioquia. Epilepsia. 1998;39(12):1334-9.

117. Pammenter MD, Rossouw EJ. Serological techniques for the diagnosis of cysticercosis. S Afr Med J. 1984;65(22):875-8.

118. Pammenter MD, Rossouw EJ. The value of an antigenic fraction of Cysticercus cellulosae in the serodiagnosis of cysticercosis. Annals of tropical medicine and parasitology. 1987;81(2):117-23.

119. Pappala BCS, Indugula JP, Shrivastava AK, Kumar S, Talabhatula SK, Kolli RS, et al. Comparative evaluation of indigenous ELISAs for detection of anti-cysticercus IgG antibodies in serum from clinically and radiologically suspected cases of neurocysticercosis. Trop Biomed. 2017;34(3):622-35.

120. Parija M, Biswas R, Harish BN, Parija SC. Detection of specific cysticercus antigen in the urine for diagnosis of neurocysticercosis. Acta tropica. 2004;92(3):253-60.

121. Parija SC, Rajesh Reddy S. Co-agglutination test for cysticercus antigen detection in the serum for the diagnosis of cysticercosis. Tropical doctor. 2006;36(3):144-7.

122. Parkhouse RME, Carpio A, Campoverde A, Sastre P, Rojas G, Cortez MM. Reciprocal contribution of clinical studies and the HP10 antigen ELISA for the diagnosis of extraparenchymal neurocysticercosis. Acta Trop. 2018;178:119-23.

123. Parkhouse RME, Carpio A, Campoverde A, Sastre P, Rojas G, Harrison LJS, et al. A modified lateral flow assay, using serum, for the rapid identification of human and bovine cysticercosis in the absence of false positives. Trans R Soc Trop Med Hyg. 2019;113(2):101-4.

124. Peralta RH, Vaz AJ, Pardini A, Macedo HW, Machado LR, De Simone SG, et al. Evaluation of an antigen from Taenia crassiceps cysticercus for the serodiagnosis of neurocysticercosis. Acta tropica. 2002;83(2):159-68.

125. Piña R, Gutiérrez AH, Gilman RH, Rueda D, Sifuentes C, Flores M, et al. A dot-ELISA using a partially purified cathepsin-L-like protein fraction from Taenia solium cysticerci, for the diagnosis of human neurocysticercosis. Ann Trop Med Parasitol. 2011;105(4):311-8.

126. Plancarte A, Fexas M, Flisser A. Reactivity in ELISA and dot blot of purified GP24, an immunodominant antigen of Taenia solium, for the diagnosis of human neurocysticercosis. International journal for parasitology. 1994;24(5):733-8.

127. Prabhakaran V, Rajshekhar V, Murrell KD, Oommen A. Taenia solium metacestode glycoproteins as diagnostic antigens for solitary cysticercus granuloma in Indian patients. Transactions of the Royal Society of Tropical Medicine and Hygiene. 2004;98(8):478-84.

128. Prabhakaran V, Rajshekhar V, Murrell KD, Oommen A. Conformation-sensitive immunoassays improve the serodiagnosis of solitary cysticercus granuloma in Indian patients. Transactions of the Royal Society of Tropical Medicine and Hygiene. 2007;101(6):570-7.

129. Proaño-Narvaez JV, Meza-Lucas A, Mata-Ruiz O, García-Jerónimo RC, Correa D. Laboratory diagnosis of human neurocysticercosis: double-blind comparison of enzyme-linked immunosorbent assay and electroimmunotransfer blot assay. Journal of clinical microbiology. 2002;40(6):2115-8.

130. Prudhivi S MS, Toleti S, Myneni RB, Rao S. Prevalence of Neurocysticercosis in and around Chinakakani, Andhra Pradesh, South India. International Journal of Current Microbiology and Applied Sciences. 2015;4(10):424-31.

131. Ramos-Kuri M, Montoya RM, Padilla A, Govezensky T, Díaz ML, Sciutto E, et al. Immunodiagnosis of neurocysticercosis. Disappointing performance of serology (enzyme-linked immunosorbent assay) in an unbiased sample of neurological patients. Archives of neurology. 1992;49(6):633-6.

132. Ribeiro Vda S, Araújo TG, Gonzaga HT, Nascimento R, Goulart LR, Costa-Cruz JM. Development of specific scFv antibodies to detect neurocysticercosis antigens and potential applications in immunodiagnosis. Immunology letters. 2013;156(1):59-67.

133. Ribeiro Vda S, Nunes Dda S, Gonzaga HT, da Cunha-Junior JP, Costa-Cruz JM. Diethylaminoethyl (DEAE) binding fraction from Taenia solium metacestode improves the neurocysticercosis serodiagnosis. Parasitology research. 2014;113(7):2569-75.

134. Ribeiro Vda S, Gonzaga HT, Nunes DDS, Goulart LR, Costa-Cruz JM. Neurocysticercosis serodiagnosis: mimotope-based synthetic peptide as potential biomarker. Parasitology research. 2019;118(5):1657-60.

135. Rodriguez S, Dorny P, Tsang VC, Pretell EJ, Brandt J, Lescano AG, et al. Detection of Taenia solium antigens and anti-T. solium antibodies in paired serum and cerebrospinal fluid samples from patients with intraparenchymal or extraparenchymal neurocysticercosis. The Journal of infectious diseases. 2009;199(9):1345-52.

136. Rodriguez-Canul R, Allan JC, Fletes C, Sutisna IP, Kapti IN, Craig PS. Comparative evaluation of purified Taenia solium glycoproteins and crude metacestode extracts by immunoblotting for the serodiagnosis of human T. solium cysticercosis. Clinical and diagnostic laboratory immunology. 1997;4(5):579-82.

137. Rosas N, Sotelo J, Nieto D. ELISA in the diagnosis of neurocysticercosis. Arch Neurol. 1986;43(4):353-6.

138. Rueda A, Sifuentes C, Gilman RH, Gutiérrez AH, Piña R, Chile N, et al. TsAg5, a Taenia solium cysticercus protein with a marginal trypsin-like activity in the diagnosis of human neurocysticercosis. Mol Biochem Parasitol. 2011;180(2):115-9.

139. Saavedra-Camacho JL, Coico-Vega MM, Failoc-Rojas VE, Ballón-Manrique B, Silva-Díaz H. Use of radiological imaging and serology by Western Blot for the diagnosis of neurocysticercosis in a hospital in northern Peru. Revista del Cuerpo Medico Hospital Nacional Almanzor Aguinaga Asenjo. 2021;14(3):311-5.

140. Sadaow L, Boonroumkaew P, Rodpai R, Janwan P, Sanpool O, Thanchomnang T, et al. Development and evaluation of an immunochromatography-based point-of-care test kit for a rapid diagnosis of human cysticercosis. Food Waterborne Parasitol. 2023;33:e00211.

141. Sahu PS, Parija SC, Narayan SK, Kumar D. Evaluation of an IgG-ELISA strategy using Taenia solium metacestode somatic and excretory-secretory antigens for diagnosis of neurocysticercosis revealing biological stage of the larvae. Acta tropica. 2009;110(1):38-45.

142. Sahu PS, Parija SC, Jayachandran S. Antibody specific to 43kDa excretory-secretory antigenic peptide of Taenia solium metacestode as a potential diagnostic marker in human neurocysticercosis. Acta tropica. 2010;115(3):257-61.

143. Sahu PS, Parija S, Kumar D, Jayachandran S, Narayan S. Comparative profile of circulating antigenic peptides in CSF, serum & urine from patients with neurocysticercosis diagnosed by immunoblotting. Parasite immunology. 2014;36(10):509-21.

144. Sahu PS, Patro S, Jena PK, Swain SK, Das BK. Imaging and Serological-Evidence of Neurocysticercosis Among Patients with Seizures in Odisha, an Unexplored Eastern Coastal Province in India. J Clin Diagn Res. 2015;9(5):Dc06-10.

145. Sako Y, Nakao M, Ikejima T, Piao XZ, Nakaya K, Ito A. Molecular characterization and diagnostic value of Taenia solium low-molecular-weight antigen genes. J Clin Microbiol. 2000;38(12):4439-44.

146. Sako Y, Takayanagui OM, Odashima NS, Ito A. Comparative Study of Paired Serum and Cerebrospinal Fluid Samples from Neurocysticercosis Patients for the Detection of Specific Antibody to Taenia solium Immunodiagnostic Antigen. Trop Med Health. 2015;43(3):171-6.

147. Salazar-Anton F, Tellez A, Lindh J. Evaluation of an immunodot blot technique for the detection of antibodies against Taenia solium larval antigens. Parasitol Res. 2012;110(6):2187-91.

148. Sanchez AL, Ljungström I, Medina MT. Diagnosis of human neurocysticerocosis in endemic countries: a clinical study in Honduras. Parasitol Int. 1999;48(1):81-9.

149. Sánchez AL, Lindbäck J, Schantz PM, Sone M, Sakai H, Medina MT, et al. A population-based, case-control study of Taenia solium taeniasis and cysticercosis. Annals of tropical medicine and parasitology. 1999;93(3):247-58.

150. Schantz PM, Sarti E, Plancarte A, Wilson M, Criales JL, Roberts J, et al. Community-based epidemiological investigations of cysticercosis due to Taenia solium: comparison of serological screening tests and clinical findings in two populations in Mexico. Clin Infect Dis. 1994;18(6):879-85.

151. Scheel CM, Khan A, Hancock K, Garcia HH, Gonzalez AE, Gilman RH, et al. Serodiagnosis of neurocysticercosis using synthetic 8-kD proteins: comparison of assay formats. The American journal of tropical medicine and hygiene. 2005;73(4):771-6.

152. Segamwenge IL, Kioko NP, Mukulu C, Jacob O, Humphrey W, Augustinus J. Neurocysticercosis among patients with first time seizure in Northern Namibia. The Pan African medical journal. 2016;24:127.

153. Shiguekawa KY, Mineo JR, de Moura LP, Costa-Cruz JM. ELISA and western blotting tests in the detection of IgG antibodies to Taenia solium metacestodes in serum samples in human neurocysticercosis. Tropical medicine & international health : TM & IH. 2000;5(6):443-9.

154. Shukla N, Husain N, Agarwal GG, Husain M. Utility of cysticercus fasciolaris antigen in Dot ELISA for the diagnosis of neurocysticercosis. Indian J Med Sci. 2008;62(6):222-7.

155. Stelzle D, Makasi CE, Schmidt V, Van Damme I, Trevisan C, Ruether C, et al. Evaluation of a point-of-care test for the diagnosis of Taenia solium neurocysticercosis in rural southern Tanzania: a diagnostic accuracy study. Lancet Infect Dis. 2024;24(1):98-106.

156. Suzuki LA, Rossi CL. Evaluation of two Taenia solium cysticercal antigenic preparations (vesicular fluid and a glycoprotein fraction with affinity for lentil lectin) for the immunodiagnosis of neurocysticercosis by enzyme-linked immunosorbent assay (ELISA). Arquivos de neuro-psiquiatria. 2011;69(3):470-4.

157. Téllez Girón E, Ramos MC, Dufour L, Montante M. [Use of the ELISA method in the diagnosis of cysticercosis]. Bol Oficina Sanit Panam. 1984;97(1):8-13.

158. Toribio L, Guzman C, Noazin S, Zimic-Sheen A, Zimic M, Gonzales I, et al. Multiantigen print immunoassay (MAPIA) for the diagnosis of neurocysticercosis: a single-center diagnostic optimization and accuracy study in Lima, Peru. J Clin Microbiol. 2023;61(12):e0076023.

159. Toribio L, Handali S, Marin Y, Perez E, Castillo Y, Bustos JA, et al. A Rapid Point-of-Care Assay for Cysticercosis Antigen Detection in Urine Samples. The American journal of tropical medicine and hygiene. 2023;108(3):578-80.

160. Tsang VC, Brand JA, Boyer AE. An enzyme-linked immunoelectrotransfer blot assay and glycoprotein antigens for diagnosing human cysticercosis (Taenia solium). J Infect Dis. 1989;159(1):50-9.

161. Vasudevan P, Moorthy RK, Rebekah G, Jackson E, Pamela BE, Thamizhmaran S, et al. Imaging correlates of serum enzyme-linked immunoelectrotransfer blot (EITB) positivity in patients with parenchymal neurocysticercosis: results from 521 patients. Transactions of the Royal Society of Tropical Medicine and Hygiene. 2022;116(2):117-23.

162. Verastegui M, Gilman RH, Garcia HH, Gonzalez AE, Arana Y, Jeri C, et al. Prevalence of antibodies to unique Taenia solium oncosphere antigens in taeniasis and human and porcine cysticercosis. The American journal of tropical medicine and hygiene. 2003;69(4):438-44.

163. Wang LN, Ge LY, Miao F, Yu ZH, Liu YB, Zhen TM, et al. [Application of EITB in immunodiagnosis of cysticercosis]. Zhongguo Ji Sheng Chong Xue Yu Ji Sheng Chong Bing Za Zhi. 2004;22(2):98-100.

164. Wilson M, Bryan RT, Fried JA, Ware DA, Schantz PM, Pilcher JB, et al. Clinical evaluation of the cysticercosis enzyme-linked immunoelectrotransfer blot in patients with neurocysticercosis. The Journal of infectious diseases. 1991;164(5):1007-9.

165. Yang HJ, Chung JY, Yun D, Kong Y, Ito A, Ma L, et al. Immunoblot analysis of a 10 kDa antigen in cyst fluid of Taenia solium metacestodes. Parasite immunology. 1998;20(10):483-8.

166. Zammarchi L, Angheben A, Fantoni T, Chiappini E, Mantella A, Galli L, et al. Screening for neurocysticercosis in internationally adopted children: yield, cost and performance of serological tests, Italy, 2001 to 2016. Euro surveillance : bulletin Europeen sur les maladies transmissibles = European communicable disease bulletin. 2018;23(40).

167. Zea-Vera A, Cordova EG, Rodriguez S, Gonzales I, Pretell EJ, Castillo Y, et al. Parasite antigen in serum predicts the presence of viable brain parasites in patients with apparently calcified cysticercosis only. Clin Infect Dis. 2013;57(7):e154-9.

168. Zimic M, Pajuelo M, Rueda D, López C, Arana Y, Castillo Y, et al. Utility of a protein fraction with cathepsin L-Like activity purified from cysticercus fluid of Taenia solium in the diagnosis of human cysticercosis. The American journal of tropical medicine and hygiene. 2009;80(6):964-70.

169. Zulu G, Stelzle, D, Mwape, KE, Van Damme, I, Trevisan, C, Mubanga, et al. The performance of a point-of-care test for the diagnosis of neurocysticercosis in a resource-poor community setting in Zambia - A diagnostic accuracy study. ssrn [Preprint]. 2024 [posted 2024 Mar 7]. Available from: https://papers.ssrn.com/sol3/Papers.cfm?abstract_id=4746924 doi: 10.2139/ssrn.4746924.
