# Supplementary material for: Accuracy of immunological tests on serum and urine for diagnosis of Taenia solium neurocysticercosis: A systematic review
Source: PLoS Negl Trop Dis. 2024 Nov 11;18(11):e0012643. doi: 10.1371/journal.pntd.0012643 (PMC11581404; doi:10.1371/journal.pntd.0012643)
Supplement: S4 Table — (DOCX) [file pntd.0012643.s008.docx]

**S4 Table. Third screening phase (full-text screening) excluded records with reason of exclusion.**

1 = Not concerning an immunological test / diagnostic reagent for NCC diagnosis; 2 = Not tested on samples of humans with confirmed NCC; 3 = Not using serum / urine as biological specimen; 4 = Insufficiently evaluating accuracy / insufficient data for accuracy calculation

| Record | Reason of exclusion |
| --- | --- |
| Arambulo 1978 [1] | 2 |
| Arroyo 2018 [2] | 4 |
| Arroyo 2022 [3] | 4 |
| Atluri 2014 [4] | 4 |
| Blocher 2011 [5] | 1 |
| Bonametti 1992 [6] | 2 |
| Bueno 2001 [7] | 2 |
| Cavalcante 2006 [8] | 4 |
| Chen 1988 [9] | 2 |
| Cho 1986 [10] | 4 |
| Coker-Vann 1984 [11] | 2 |
| Corda 2022 [12] | 4 |
| Correa 1999 [13] | 2 |
| Costa 1986 [14] | 2 |
| Dao 1973 [15] | 2 |
| Dekumyoy 1998 [16] | 2 |
| Diwan 1982 [17] | 2 |
| Erhart 2002 [18] | 4 |
| Ev 1999 [19] | 4 |
| Fleury 2013 [20] | 4 |
| Furrows 2006 [21] | 2 |
| Garcia 1994 [22] | 1 |
| Gevorkian 1996 [23] | 2 |
| Grogl 1985 [24] | 4 |
| Hancock 2003 [25] | 2 |
| Hancock 2004 [26] | 2 |
| Hernández-Cruz 2009 [27] | 2 |
| Husain 2008 [28] | 4 |
| Ikejima 2005 [29] | 2 |
| Ito 1998 [30] | 2 |
| Jiang 1990 [31] | 2 |
| Jiang 1991 [32] | 2 |
| Kim 1986 [33] | 2 |
| Ko 1998a [34] | 2 |
| Ko 1998b [35] | 4 |
| Lin 2022 [36] | 2 |
| Liu 2001 [37] | 2 |
| Manhani 2009 [38] | 1 |
| Marlet 1978 [39] | 3 |
| McCleery 2020 [40] | 4 |
| Miranda-Ulloa 2014 [41] | 2 |
| Mondragón1994 [42] | 4 |
| Morakote 1986 [43] | 2 |
| Mubanga 2021 [44] | 2 |
| Mwape 2011 [45] | 2 |
| Nascimento 1984 [46] | 2 |
| Nhancupe 2016 [47] | 2 |
| Paredes 2016 [48] | 4 |
| Pialarissi 1995 [49] | 3 |
| Plancarte 1987 [50] | 2 |
| Plancarte 1999 [51] | 4 |
| Prado-Jean 2007 [52] | 2 |
| Praet 2010 [53] | 2 |
| Romo 2020 [54] | 4 |
| Sako 2013 [55] | 2 |
| Salazar-Anton 2012 [56] | 2 |
| Sato 2006 [57] | 2 |
| Simac 1995 [58] | 4 |
| Sloan 1995 [59] | 2 |
| Villota 2003 [60] | 2 |
| Yong 1993 [61] | 2 |
| Zhang 2016 [62] | 2 |
| Zini 1990 [63] | 4 |

**References**

1. Arambulo PV, 3rd, Walls KW, Bullock S, Kagan IG. Serodiagnosis of human cysticercosis by microplate enzyme-linked immunospecific assay (ELISA). Acta tropica. 1978;35(1):63-7.

2. Arroyo G, Rodriguez S, Lescano AG, Alroy KA, Bustos JA, Santivañez S, et al. Antibody Banding Patterns of the Enzyme-Linked Immunoelectrotransfer Blot and Brain Imaging Findings in Patients With Neurocysticercosis. Clin Infect Dis. 2018;66(2):282-8.

3. Arroyo G, Bustos JA, Lescano AG, Gonzales I, Saavedra H, Pretell EJ, et al. Improved Diagnosis of Viable Parenchymal Neurocysticercosis by Combining Antibody Banding Patterns on Enzyme-Linked Immunoelectrotransfer Blot (EITB) with Antigen Enzyme-Linked Immunosorbent Assay (ELISA). J Clin Microbiol. 2022;60(2):e0155021.

4. Atluri VS, Gogulamudi VR, Singhi P, Khandelwal N, Parasa LS, Malla N. Pediatric neurocysticercosis: usefulness of antibody response in cysticidal treatment follow-up. BioMed research international. 2014;2014:904046.

5. Blocher J, Schmutzhard E, Wilkins PP, Gupton PN, Schaffert M, Auer H, et al. A cross-sectional study of people with epilepsy and neurocysticercosis in Tanzania: clinical characteristics and diagnostic approaches. PLoS neglected tropical diseases. 2011;5(6):e1185.

6. Bonametti AM, Basile MA, Vaz AJ, Baldy JL, Takiguti CK. [The positivity index of the immunoenzyme reaction (ELISA) for cysticercosis in the cerebrospinal fluid (CSF) and in the serum of epilepsy patients]. Rev Inst Med Trop Sao Paulo. 1992;34(5):451-8.

7. Bueno EC, Snege M, Vaz AJ, Leser PG. Serodiagnosis of human cysticercosis by using antigens from vesicular fluid of Taenia crassiceps cysticerci. Clinical and diagnostic laboratory immunology. 2001;8(6):1140-4.

8. Cavalcante MMFP CJ, Costa-Cruz JM, Barbosa AP, Silva SA, Campos DMB. Clinical, epidemiological and laboratory criteria for the diagnosis of human cysticercosis in Brazilian patients. Acta Parasitologica. 2006;51(1):79-81.

9. Chen JP, Zhang XY, Liu MF, Xiong J, Feng ML, Hu RY, et al. [Evaluation of Cysticercus cellulosae antigens in the immunodiagnosis of human cysticercosis by ELISA]. Zhongguo ji sheng chong xue yu ji sheng chong bing za zhi = Chinese journal of parasitology & parasitic diseases. 1988;6(1):32-5.

10. Cho SY, Kim SI, Kang SY. Serologic follow-up study in neurocysticercosis patients by ELISA after praziquantel treatment. Kisaengchunghak Chapchi. 1986;24(2):159-70.

11. Coker-Vann M, Brown P, Gajdusek DC. Serodiagnosis of human cysticercosis using a chromatofocused antigenic preparation of Taenia solium cysticerci in an enzyme-linked immunosorbent assay (ELISA). Transactions of the Royal Society of Tropical Medicine and Hygiene. 1984;78(4):492-6.

12. Corda M, Sciurba J, Blaha J, Mahanty S, Paredes A, Garcia HH, et al. A recombinant monoclonal-based Taenia antigen assay that reflects disease activity in extra-parenchymal neurocysticercosis. PLoS neglected tropical diseases. 2022;16(5):e0010442.

13. Correa D, Sarti E, Tapia-Romero R, Rico R, Alcántara-Anguiano I, Salgado A, et al. Antigens and antibodies in sera from human cases of epilepsy or taeniasis from an area of Mexico where Taenia solium cysticercosis is endemic. Ann Trop Med Parasitol. 1999;93(1):69-74.

14. Costa JM. [Immunoenzymatic test (ELISA) in the diagnosis of neurocysticercosis: study of various antigenic extracts in the detection of IgG antibodies in serum and cerebrospinal fluid samples]. Arquivos de neuro-psiquiatria. 1986;44(1):15-31.

15. Dao C, Arnaud JP, Petithory J, Brumpt L. The immunological diagnosis of human cysticercosis by the indirect fluorescent technique (First results). Annales de Parasitologie Humaine et Comparee. 1973;48(1):23-32.

16. Dekumyoy P, Vanijanonta S, Waikagul J, Sa-nguankiat S, Danis M. Use of delipidized antigens of Taenia solium metacestodes in IgG-ELISA for detection of neurocysticercosis. The Southeast Asian journal of tropical medicine and public health. 1998;29(3):572-8.

17. Diwan AR, Coker-Vann M, Brown P, Subianto DB, Yolken R, Desowitz R, et al. Enzyme-linked immunosorbent assay (ELISA) for the detection of antibody to cysticerci of Taenia solium. The American journal of tropical medicine and hygiene. 1982;31(2):364-9.

18. Erhart A, Dorny P, Van De N, Vien HV, Thach DC, Toan ND, et al. Taenia solium cysticercosis in a village in northern Viet Nam: seroprevalence study using an ELISA for detecting circulating antigen. Trans R Soc Trop Med Hyg. 2002;96(3):270-2.

19. Ev LV, Maia AA, Pianetti G, Nascimento E. Immunological evaluation of a 26-kDa antigen from Taenia solium larvae for specific immunodiagnosis of human neurocysticercosis. Parasitology research. 1999;85(2):98-102.

20. Fleury A, Garcia E, Hernández M, Carrillo R, Govezensky T, Fragoso G, et al. Neurocysticercosis: HP10 antigen detection is useful for the follow-up of the severe patients. PLoS neglected tropical diseases. 2013;7(3):e2096.

21. Furrows SJ, McCroddan J, Bligh WJ, Chiodini P. Lack of specificity of a single positive 50-kDa band in the electroimmunotransfer blot (EITB) assay for cysticercosis. Clin Microbiol Infect. 2006;12(5):459-62.

22. Garcia HH, Herrera G, Gilman RH, Tsang VC, Pilcher JB, Diaz JF, et al. Discrepancies between cerebral computed tomography and western blot in the diagnosis of neurocysticercosis. The Cysticercosis Working Group in Peru (Clinical Studies Coordination Board). The American journal of tropical medicine and hygiene. 1994;50(2):152-7.

23. Gevorkian G, Manoutcharian K, Larralde C, Hernandez M, Almagro JC, Viveros M, et al. Immunodominant synthetic peptides of Taenia crassiceps in murine and human cysticercosis. Immunology letters. 1996;49(3):185-9.

24. Grogl M, Estrada JJ, MacDonald G, Kuhn RE. Antigen-antibody analyses in neurocysticercosis. J Parasitol. 1985;71(4):433-42.

25. Hancock K, Khan A, Williams FB, Yushak ML, Pattabhi S, Noh J, et al. Characterization of the 8-kilodalton antigens of Taenia solium metacestodes and evaluation of their use in an enzyme-linked immunosorbent assay for serodiagnosis. Journal of Clinical Microbiology. 2003;41(6):2577-86.

26. Hancock K, Pattabhi S, Greene RM, Yushak ML, Williams F, Khan A, et al. Characterization and cloning of GP50, a Taenia solium antigen diagnostic for cysticercosis. Molecular and Biochemical Parasitology. 2004;133(1):115-24.

27. Hernández-Cruz E, González-Cabriales JJ, Ordaz-Pichardo C, de la Cruz-Hernández NI, Flores-Gutiérrez GH. Development of an immunobinding dot-blot assay as an alternative for the serodiagnosis of human cysticercosis. Journal of helminthology. 2009;83(4):333-7.

28. Husain N, Shukla N, Kumar R, Husain M, Chaturvedi A, Agarwal GG, et al. ELISA in the evaluation of therapeutic response to albendazole in neurocysticercosis. The Journal of infection. 2008;56(1):65-73.

29. Ikejima T, Piao ZX, Sako Y, Sato MO, Bao S, Si R, et al. Evaluation of clinical and serological data from Taenia solium cysticercosis patients in eastern Inner Mongolia Autonomous Region, China. Trans R Soc Trop Med Hyg. 2005;99(8):625-30.

30. Ito A, Plancarte A, Ma L, Kong Y, Flisser A, Cho SY, et al. Novel antigens for neurocysticercosis: simple method for preparation and evaluation for serodiagnosis. The American journal of tropical medicine and hygiene. 1998;59(2):291-4.

31. Jiang HJ, Yang LX, Pang SH, Sun T, Xie SP, Meng JM, et al. [Dot-ELISA in the diagnosis of neurocysticercosis]. Zhongguo ji sheng chong xue yu ji sheng chong bing za zhi = Chinese journal of parasitology & parasitic diseases. 1990;8(3):207-9.

32. Jiang HJ, Bian YH, Wang FY. Diagnosis of neurocysticercosis by immunoenzymatic staining technique (IEST) with frozen sections of Cysticercus cellulosae. Zhongguo ji sheng chong xue yu ji sheng chong bing za zhi = Chinese journal of parasitology & parasitic diseases. 1991;9(1):65-7.

33. Kim SI, Kang SY, Cho SY, Hwang ES, Cha CY. Purification of cystic fluid antigen of Taenia solium metacestodes by affinity chromatography using monoclonal antibody and its antigenic characterization. Kisaengch'unghak chapchi The Korean journal of parasitology. 1986;24(2):145-8.

34. Ko RC, Ng TF. Evaluation of excretory/secretory products of larval Taenia solium as diagnostic antigens for porcine and human cysticercosis. Journal of helminthology. 1998;72(2):147-54.

35. Ko RC, Ng TF. Specificity of isoelectric focusing-purified antigens in the diagnosis of human cysticercosis. Parasitology research. 1998;84(7):565-9.

36. Lin XG, Jiang Y, Wu JJ, Eda S, Wan N. An alternating current electrokinetics biosensor for rapid on-site serological screening of Taenia solium cysticercosis infection. Microchimica acta. 2022;189(12).

37. Liu ZP, Li H, Zhu LY, Liu S, Xue HC. [Application of dot immunogold filtration assay in antibody detection for cysticercosis]. Zhongguo ji sheng chong xue yu ji sheng chong bing za zhi = Chinese journal of parasitology & parasitic diseases. 2001;19(6):354-6.

38. Manhani MN, da Silva Ribeiro V, Oliveira Silva DA, Costa-Cruz JM. Specific IgG avidity in active and inactive human neurocysticercosis. Diagnostic Microbiology and Infectious Disease. 2009;65(2):211-3.

39. Marlet JM. [An intradermal antigen for the diagnosis of cysticercosis]. Rev Saude Publica. 1978;12(1):23-5.

40. McCleery E, Allen SE, Moyano LM, Gamboa R, Vilchez P, Muro C, et al. Population Screening for Urine Antigens to Detect Asymptomatic Subarachnoid Neurocysticercosis: A Pilot Study in Northern Peru. The American journal of tropical medicine and hygiene. 2020;103(3):1125-8.

41. Miranda-Ulloa E, Sandoval-Ahumada R, Ayala E, Vásquez-Ampuero J. [Assessment of dot blot tests and latex agglutination for cysticercosis diagnostic in Peru]. Revista peruana de medicina experimental y salud publica. 2014;31(2):297-301.

42. Mondragón A, Plancarte A, Flisser A. [Diagnosis of human cysticercosis with ELISA]. Salud publica de Mexico. 1994;36(4):393-8.

43. Morakote N, Charuchinda K, Thammasonthi W, Khamboonruang C. Evaluation of counterimmunoelectrophoresis for serodiagnosis of human cysticercosis. The Southeast Asian journal of tropical medicine and public health. 1986;17(4):537-42.

44. Mubanga C, Van Damme I, Trevisan C, Schmidt V, Phiri IK, Zulu G, et al. Evaluation of an Antibody Detecting Point of Care Test for Diagnosis of Taenia solium Cysticercosis in a Zambian Rural Community: A Prospective Diagnostic Accuracy Study. Diagnostics (Basel, Switzerland). 2021;11(11).

45. Mwape KE, Praet N, Benitez-Ortiz W, Muma JB, Zulu G, Celi-Erazo M, et al. Field evaluation of urine antigen detection for diagnosis of Taenia solium cysticercosis. Transactions of the Royal Society of Tropical Medicine and Hygiene. 2011;105(10):574-8.

46. Nascimento E, Mayrink W. [Evaluation of Cysticercus cellulosae antigens in the immunodiagnosis of human cysticercosis by indirect hemagglutination]. Revista do Instituto de Medicina Tropical de Sao Paulo. 1984;26(6):289-94.

47. Nhancupe N, Noormahomed EV, Afonso S, Falk KI, Lindh J. Performance of Tsol-p27 antigen for the serological diagnosis of cysticercosis in Mozambique. Journal of helminthology. 2016;90(5):630-3.

48. Paredes A, Sáenz P, Marzal MW, Orrego MA, Castillo Y, Rivera A, et al. Anti-Taenia solium monoclonal antibodies for the detection of parasite antigens in body fluids from patients with neurocysticercosis. Experimental parasitology. 2016;166:37-43.

49. Pialarissi CS, Nitrini SM. [Comparison between the erythroimmunoadsorption test by capture, immunoenzymatic and passive hemagglutination tests used in the diagnosis of neurocysticercosis]. Revista de saude publica. 1995;29(2):115-9.

50. Plancarte A, Espinoza B, Flisser A. Immunodiagnosis of human neurocysticercosis by enzyme-linked immunosorbent assay. Child's nervous system : ChNS : official journal of the International Society for Pediatric Neurosurgery. 1987;3(4):203-5.

51. Plancarte A, Hirota C, Martínez-Ocaña J, Mendoza-Hernández G, Zenteno E, Flisser A. Characterization of GP39-42 and GP24 antigens from Taenia solium cysticerci and of their antigenic GP10 subunit. Parasitology research. 1999;85(8):680-4.

52. Prado-Jean A, Kanobana K, Druet-Cabanac M, Nsengyiumva G, Dorny P, Preux PM, et al. Combined use of an antigen and antibody detection enzyme-linked immunosorbent assay for cysticercosis as tools in an epidemiological study of epilepsy in Burundi. Trop Med Int Health. 2007;12(7):895-901.

53. Praet N, Rodriguez-Hidalgo R, Speybroeck N, Ahounou S, Benitez-Ortiz W, Berkvens D, et al. Infection with versus Exposure to Taenia solium: What Do Serological Test Results Tell Us? American journal of tropical medicine and hygiene. 2010;83(2):413-5.

54. Romo ML, Hernández M, Astudillo OG, Diego G, de-la-Rosa-Arana JL, Meza-Lucas A, et al. Diagnostic value of glycoprotein band patterns of three serologic enzyme-linked immunoelectrotransfer blot assays for neurocysticercosis. Parasitol Res. 2020;119(8):2521-9.

55. Sako Y, Itoh S, Okamoto M, Nakaya K, Ito A. Simple and reliable preparation of immunodiagnostic antigens for Taenia solium cysticercosis. Parasitology. 2013;140(13):1589-94.

56. Salazar-Anton F, Tellez A, Lindh J. Evaluation of an immunodot blot technique for the detection of antibodies against Taenia solium larval antigens. Parasitology research. 2012;110(6):2187-91.

57. Sato MO, Sako Y, Nakao M, Yamasaki H, Nakaya K, Ito A. Evaluation of purified Taenia solium glycoproteins and recombinant antigens in the serologic detection of human and swine cysticercosis. The Journal of infectious diseases. 2006;194(12):1783-90.

58. Simac C, Michel P, Andriantsimahavandy A, Esterre P, Michault A. Use of enzyme-linked immunosorbent assay and enzyme-linked immunoelectrotransfer blot for the diagnosis and monitoring of neurocysticercosis. Parasitology research. 1995;81(2):132-6.

59. Sloan L, Schneider S, Rosenblatt J. Evaluation of enzyme-linked immunoassay for serological diagnosis of cysticercosis. Journal of clinical microbiology. 1995;33(12):3124-8.

60. Villota GE, Gomez DI, Volcy M, Franco AF, Cardona EA, Isaza R, et al. Similar diagnostic performance for neurocysticercosis of three glycoprotein preparations from Taenia solium metacestodes. The American journal of tropical medicine and hygiene. 2003;68(3):276-80.

61. Yong TS, Yeo IS, Seo JH, Chang JK, Lee JS, Kim TS, et al. Serodiagnosis of cysticercosis by ELISA-inhibition test using monoclonal antibodies. The Korean journal of parasitology. 1993;31(2):149-56.

62. Zhang Y, Bae YA, Zong HY, Kong Y, Cai GB. Functionally Expression of Metalloproteinase in Taenia solium Metacestode and Its Evaluation for Serodiagnosis of Cysticercosis. Iranian journal of parasitology. 2016;11(1):35-45.

63. Zini D, Farrell VJ, Wadee AA. The relationship of antibody levels to the clinical spectrum of human neurocysticercosis. J Neurol Neurosurg Psychiatry. 1990;53(8):656-61.
