# Supplementary material for: Accuracy of immunological tests on serum and urine for diagnosis of Taenia solium neurocysticercosis: A systematic review
Source: PLoS Negl Trop Dis. 2024 Nov 11;18(11):e0012643. doi: 10.1371/journal.pntd.0012643 (PMC11581404; doi:10.1371/journal.pntd.0012643)
Supplement: Attachment — Submitted filename: Comments PNTD-D-24-00877.docx [file pntd.0012643.s010.docx]

Dr. Acker and colleagues have conducted a systematic review to summarize the evidence on the validity of serum/urine immunoassays versus neuroimaging and/or surgery/biopsy as reference standards in the diagnosis of neurocysticercosis (NCC), focusing on cyst location, stage and number. The authors have (1) reported a significant proportion of poor-quality articles in several domains of the QUADAS-2 tool; (2) noted the substantial heterogeneity of data, which limited the performance of a meta-analysis; (3) highlighted the lack of data reported in the articles, and the challenges of interpreting and comparing the validity results of immunodiagnostic tests; (4) innovated by proposing some information to be taken into account to improve the STARD 2015 reporting guideline for diagnostic accuracy studies assessing immunodiagnosis of NCC; and (5) documented some immunological tests that showed "good" sensitivity or specificity estimates according to certain cyst characteristics (location, stage or stage). I have the following comments:

**Major comments**

1. With the exception of the selection of titles and abstracts for English articles which was carried out by two authors, **all other screening phases, as well as data extraction and study quality assessment, were carried out by a single author**. The authors mention that data collection underwent quality control by a second reviewer only for **10%** of English records without more information on the results. "Best practice guidelines for conducting systematic reviews argue that the literature search and sifting process is ideally conducted by two separate reviewers, who must both agree on work to be included." (Siddaway et al., 2019; PMID: 30089228). Given the complexity and diversity/abundance of information reported, this raises serious questions about the quality/validity of the data presented in this review and constitutes a major limitation of the study. A second assessment of the records by another author would increase the credibility of the results.
2. In assessing study quality, a higher proportion of articles with high or unclear risk of bias was reported on several signaling questions (Q) in all domains of the QUADAS-2 tool: participant selection (Q1, Q2 and Q4), index test (Q6 and Q7), reference standard (Q10 and Q11), and flow and timing (Q14 and Q15). The discussion should highlight the poor quality of most studies and call for a cautious interpretation of reported sensitivity and specificity estimates, **including those presented as a “good performance”**.
3. The assessment of study quality using the QUADAS-2 tool focuses not only on bias, but also on the applicability of eligible studies for the first three QUADAS-2 domains. Why didn't the authors assess the applicability of eligible studies to their review?
4. The authors mention having assigned a level of certainty (either 'definite', or 'probable', or 'possible') to each evaluated entity on the basis **predefined criteria**. Such criteria should be clearly presented and referenced to increase process transparency.
5. The review have included studies that used neuroimaging and/or surgery/biopsy as reference standards as the reference test. First, given that a test such as neuroimaging may be more imperfect than surgery/biopsy, it is essential that the specific reference test applied in each study be indicated (add a column in Tables 1 and 2). Furthermore, assuming that neuroimaging without surgery/biopsy would predominate as the reference standard, did the studies often apply statistical methods that can account for misclassification errors associated with the imperfection of both index test and reference standard (e.g. Bayesian latent class models)? This would be worth discussing.
6. The manuscript deals with a very important subject, however, the data presented are so diverse that it may be difficult for an epidemiologist or clinician to derive any specific information. The writing of the article can be improved, and an additional effort in synthesis could be made, in particular, for the results (e.g. the 'data analysis' section) and the discussion, which are rather long.
7. All figures are of very poor quality and need to be improved.
8. Include a paragraph on the limitations of the study.

**Minor comments**

9. **Introduction, Lines 103-105**: Among the challenges of neuroimaging, one should mention the technical challenges associated with image quality, which depend in particular on the performance (high/low resolution) of the machines used. Another issue may be that some radiographic images of NCC are non-specific and can be seen in other conditions (Kimura-Hayama et al., 2010; PMID: 21071384).
10. **Methods, Lines 141-143**: Specify that the second question of the review refers to the performance of immunological tests.
11. **Methods, Lines 165-167**: How should the fact that the review is interested in the location, stage and number of cysts limit the inclusion of articles that have only reported the specificity of the test?
12. **Table 1**: For parenchymal active multiple cysts, ab ELISA results show extremely low sensitivity (several zeros) for T. solium somatic Ag: WCE and T. solium synthetic Ag reagents. Is there a specific explanation for this? This could be highlighted in the discussion.
13. **Figure 2**: Specify the number of studies for each exclusion criterion for the second screening phase. Indicate also the number of records submitted but not yet published (n=1?)
