# Supplementary material for: Accuracy of immunological tests on serum and urine for diagnosis of Taenia solium neurocysticercosis: A systematic review
Source: PLoS Negl Trop Dis. 2024 Nov 11;18(11):e0012643. doi: 10.1371/journal.pntd.0012643 (PMC11581404; doi:10.1371/journal.pntd.0012643)
Supplement: Attachment — Submitted filename: Response to reviewers.docx [file pntd.0012643.s011.docx]

**Response to reviewers’ comments:**

**Major comments**

1. With the exception of the selection of titles and abstracts for English articles which was carried out by two authors, **all other screening phases, as well as data extraction and study quality assessment, were carried out by a single author**. The authors mention that data collection underwent quality control by a second reviewer only for **10%** of English records without more information on the results. "Best practice guidelines for conducting systematic reviews argue that the literature search and sifting process is ideally conducted by two separate reviewers, who must both agree on work to be included." (Siddaway et al., 2019; PMID: 30089228). Given the complexity and diversity/abundance of information reported, this raises serious questions about the quality/validity of the data presented in this review and constitutes a major limitation of the study. A second assessment of the records by another author would increase the credibility of the results.

Response: Thank you for this comment. We indeed understand the concerns you are addressing. The title and abstract (TIAB) screening of English articles was performed by two authors, ensuring a solid basis for further screening. Any discrepancies were thoroughly discussed and resolved before making a final decision for inclusion/exclusion in this phase. Unfortunately, due to limited available time and resources there was no option to carry out the full-text screening, data collection or study quality assessment by more than one author per language. However, authors that were asked to perform abovementioned processes where all very acquainted with the research topic and review design, and a detailed instruction sheet for each process was developed by the lead author (LVA) and provided for the co-authors (LT, HZ, MC) with sufficient explanation, allowing a similar and consistent approach. All received data was double-checked for missing information and unclarities by the lead author (LVA) before further processing.

Regarding the quality control performed, control of 10% of English articles was deemed sufficient to reach the intended aim, i.e. to evaluate the reliability of the data extraction process based on developed instruction sheet. Discrepancies were documented and evaluated by the two authors (LVA, MC) (no consensus with a third author needed). No systematic errors in data extraction were perceived, suggesting reliable data extraction in previous process, and not requiring modification of the used methods as described in the developed instruction sheet. In the manuscript, this was added in lines 251-253 of the Results-Search results section, as follows:

*“Of 159 English records, 10% (corresponding to 16 records) were randomly selected for quality control to evaluate data extraction reliability. No systematic errors were perceived in the data collection process.”*

The issue is further also mentioned in an added paragraph on the limitations of the study (see question 8).

2. In assessing study quality, a higher proportion of articles with high or unclear risk of bias was reported on several signaling questions (Q) in all domains of the QUADAS-2 tool: participant selection (Q1, Q2 and Q4), index test (Q6 and Q7), reference standard (Q10 and Q11), and flow and timing (Q14 and Q15). The discussion should highlight the poor quality of most studies and call for a cautious interpretation of reported sensitivity and specificity estimates, **including those presented as a “good performance”**.

Response: Thank you for indicating this. We agree the identified bias is an important matter to address. We have added the following sentences to the Discussion section, lines 462-465:

*“Also, the RoB assessment of this review demonstrates that many studies likely carry bias, either in patient selection, in index testing, reference standard testing, or in flow and timing. Reported test results should therefore always be interpreted attentively, including highly promising results.”*

3. The assessment of study quality using the QUADAS-2 tool focuses not only on bias, but also on the applicability of eligible studies for the first three QUADAS-2 domains. Why didn't the authors assess the applicability of eligible studies to their review?

Response: Thank you for this question. The QUADAS-2 RoB assessment indeed normally also includes an assessment of the applicability of eligible studies. Applicability judgements refer to the review question, in assessment whether the study results help answer this question, i.e. whether study participants are similar to those defined in the research question and whether used index test and reference standard match those defined in the research question. Our review questions do not mention specific requirements for patients, index tests or used reference standard. Our defined eligibility criteria do set certain minimal criteria regarding patient selection, used index test and reference standard. Thus, all included studies for RoB assessment eventually fulfilled these criteria which, according to the authors, were meaningful to address the review questions.

4. The authors mention having assigned a level of certainty (either 'definite', or 'probable', or 'possible') to each evaluated entity on the basis **predefined criteria**. Such criteria should be clearly presented and referenced to increase process transparency.

Response: Thank you. The mentioned ‘predefined criteria’ refer to the criteria as displayed in Fig 1 (e.g. criteria for certainty level ‘probable’ regarding parenchymal cyst localization are “single cyst granuloma (SCG), any reference to inactive cyst phase”). Criteria were developed according to authors’ decision. We agree that this could be phrased more clearly in the manuscript text, and have made following adaptations, in lines 206-208:

“Based on *developed criteria as can be consulted in Fig 1*, a level of certainty (either ‘definite’, or ‘probable’, or ‘possible’) was assigned to each evaluated entity, regarding both cyst localization and stage.”

5. The review have included studies that used neuroimaging and/or surgery/biopsy as reference standards as the reference test. First, given that a test such as neuroimaging may be more imperfect than surgery/biopsy, it is essential that the specific reference test applied in each study be indicated (add a column in Tables 1 and 2). Furthermore, assuming that neuroimaging without surgery/biopsy would predominate as the reference standard, did the studies often apply statistical methods that can account for misclassification errors associated with the imperfection of both index test and reference standard (e.g. Bayesian latent class models)? This would be worth discussing.

Response: Thank you for indicating this necessary adaptation. We have added a column ‘RS’ ( = reference standard) to Tables 1 and 2, indicating the used reference standard (i.e. CT, MRI, imaging (not further specified), NS (not specified but data on cyst localization and phase available), surgery/biopsy).

Furthermore, we appreciate that you have raised an important point concerning the potential limitations in the reference standards used in the included studies. It is correct that neuroimaging could potentially be a less definitive reference standard than surgery/biopsy and could therefore introduce misclassification errors. We added a brief section to the Discussion section highlighting this limitation to be considered in future studies.

Lines 510-514: “*Another key limitation is the variability in reference standards across included studies. While neuroimaging is commonly used, it can not be truly considered as a gold standard technique, potentially leading to misclassification errors. We did not evaluate if included studies applied methods (e.g. Bayesian latent class models) to address potential errors. Future reviews could consider this aspect to strengthen conclusions*.”

6. The manuscript deals with a very important subject, however, the data presented are so diverse that it may be difficult for an epidemiologist or clinician to derive any specific information. The writing of the article can be improved, and an additional effort in synthesis could be made, in particular, for the results (e.g. the 'data analysis' section) and the discussion, which are rather long.

Response: Thank you for this suggestion. We tried to clarify and make it understandable for readers. We modified some sections, as suggested:

1) The Results-Data analysis section was adapted as follows, to improve readability:

Lines 276-280: Data classification was performed to select studies providing adequate data on cyst localization and cyst stage*~~, making test accuracy results usable for further analysis~~*. Of 169 records included for data collection, corresponding to 504 evaluated tests -as some records evaluate more than one index test-, 116 records *~~and 381 tests, respectively,~~* corresponding to 381 tests were excluded according to exclusion criteria (Fig 4).

Line 285: “South Asia ~~(e.g. India)~~”

Line 286: “East Asia ~~(e.g. China, South Korea)~~”

Lines 288-293: “~~For each evaluated test, the reported test results were included with high confidence (Table 1), both high and low confidence (Tables 1 and 2), or low confidence alone (Table 2). Corresponding to these confidence levels, 89, 6, and 8 tests were included in the category of serological antibody detection, and 14, 1 and 0 were included in the category of serological antigen detection. All three tests in the category of urine-based antibody detection, as well as the two tests in the category of urine-based antigen detection, were included with high confidence.~~”

Lines 299-332: The order in which results are discussed was adapted to match the order in which tests are ranked in Table 1.

Lines 307-311: “The newly developed multi-antigen print immunoassay (MAPIA) based on  ~~a combination of the recombinant forms of LLGP (rGP50, rT24H and sTs14)~~ *three recombinant and synthetic antigens,* ~~stands out for detection of both~~ *shows notable sensitivity for* parenchymal active single and multiple cysts (100.0%) and extraparenchymal cysts in the subarachnoid space (100.0%), and delivers high specificity ~~results~~ (98.5%) [31]. Although *these* results are ~~for now~~ based on small sample sizes, the MAPIA has potential to form an interesting addition to standard laboratory performed tests.”

Lines 318-319: “Similarly, urine-based antigen-detecting tests (antigen ELISA and POC dipstick ~~test formats~~)”

Lines 345-347: “One describes a ~~proof-of-concept for a~~ urine-based dipstick assay with laboratory-based ~~test~~ evaluation of samples from ~~individuals with~~ subarachnoid NCC *patients*”

2) The Discussion section (lines 378-517) was adapted as follows: Many small adaptations were made to improve the readability of this section and to shorten it, however no substantial changes were made to the discussion content. For these adaptations we refer to the manuscript document with tracked changes.
7. All figures are of very poor quality and need to be improved.

Response: Thank you for pointing this out. The figures were adapted to better quality.

8. Include a paragraph on the limitations of the study.

Response: We would like to thank you for pointing out the necessity for this extra paragraph. The following text was added at the end of the Discussion section (lines 501-517):

*“Our study had some important limitations. Some publication bias may have been introduced, as mainly published records were screened. The full-text screening, data collection and RoB assessment were performed by one author per language only due to time and resource limitations. Yet for data collection, a 10% quality control of English articles was completed. The QUADAS-2 RoB assessment was performed only assessing risk of bias and not applicability of eligible studies, as we believed our eligibility criteria were robust enough to exclude those articles not aligning with the review questions. To maintain a sufficient level of confidence in cyst characteristics data, confidence scaling was done according to authors’ predeveloped criteria, resulting in exclusion of many records and corresponding tests. As such, accuracy data might have been skewed and this must be considered when consulting the results. Another key limitation is the variability in reference standards across included studies. While neuroimaging is commonly used, it can not be truly considered as a gold standard technique, potentially leading to misclassification errors. We did not evaluate if included studies applied methods (e.g. Bayesian latent class models) to address potential errors. Future reviews could consider this aspect to strengthen conclusions*. *Lastly, this review has highlighted that interpretation and comparison of test results was highly challenging due to data heterogeneity, impeding the possibility to perform meta-analysis. Nevertheless, the narrative analysis effectively elucidates immunological test performance across clinically relevant scenarios.”*

**Minor comments**

9. **Introduction, Lines 103-105**: Among the challenges of neuroimaging, one should mention the technical challenges associated with image quality, which depend in particular on the performance (high/low resolution) of the machines used. Another issue may be that some radiographic images of NCC are non-specific and can be seen in other conditions (Kimura-Hayama et al., 2010; PMID: 21071384).

Response: Thank you for this useful addition. We have added your suggestions to the Introduction and Discussion section.

Lines 104-106: “Also, the use of these modalities is costly, and requires highly-trained staff *capable of interpreting images of varying quality depending on the obtainable image resolution* [13].

Lines 437-440: *“*Imaging scans can display typical pathognomonic lesions for NCC, but may also be limited to showing highly suggestive lesions or compatible lesions *not clearly discernible from lesions caused by other conditions* [11].”

10. **Methods, Lines 141-143**: Specify that the second question of the review refers to the performance of immunological tests.

Response: Thank you, this will indeed clarify the second research question. We have made the following adaptation in the text (lines 144-146):

“What is the performed accuracy *of immunological tests* for diagnosis of neurocysticercosis with different cyst localization, stage, and number?”

11. **Methods, Lines 165-167**: How should the fact that the review is interested in the location, stage and number of cysts limit the inclusion of articles that have only reported the specificity of the test?

Response: Thank you for this question. Given our interest in the localization, stage and number of cysts, we prioritized studies that provided detailed information on these characteristics. Articles only reporting on test specificity without considering these cyst characteristics did not offer the comprehensive data needed for assessment of test performance in different clinical scenarios, and therefore not contributing to answer the second research question. We therefore excluded these from the review.

12. **Table 1**: For parenchymal active multiple cysts, ab ELISA results show extremely low sensitivity (several zeros) for T. solium somatic Ag: WCE and T. solium synthetic Ag reagents. Is there a specific explanation for this? This could be highlighted in the discussion.

Response: Thank you for this remark. We presume you are referring to the sensitivity results of ab ELISA for parenchymal *inactive* multiple cysts, which indeed show several low results.

Regarding *T. solium* somatic Ag WCE: Atluri et al. 2009b refers to a small sample size for patients with calcified (i.e. inactive) cysts, making it difficult to draw conclusions. Schantz 1994 there is also a low sample size of patients with inactive multiple cysts; this is not further discussed.

Regarding *T. solium* synthetic Ag: All data in this category is derived from a record by Ferrer et al. 2012. This record states that “surprisingly, neither the Ts8B2 peptides nor the CyDA peptides were recognised by infected humans” unlike recombinant rTs8B2 proteins, and that “this differential antigenicity of 8kDa family peptides could be related with parasite antigenic variability” “among different parasite isolates”.

As the results are of detection of inactive cysts, we find them less relevant to elaborately discuss in the Discussion section. However in the Introduction section lines 116-117 we state that “crude antigens do not score well on diagnostic performance”, and in the Results-Data analysis section lines 306-307 we state that “Regarding the use of recombinant and/or synthetic antigens as test diagnostic reagents, results were variable”. In the Discussion lines 450-451, it is mentioned that “We must always hold into account that test performance results are highly setting-specific, and can not be plainly extrapolated.”

13. **Figure 2**: Specify the number of studies for each exclusion criterion for the second screening phase. Indicate also the number of records submitted but not yet published (n=1?)

Response: Thank you. The second screening phase (i.e. TIAB-screening) covered the screening of 915 records. In this screening phase, records often matched more than one exclusion criterion, and screening was performed by multiple authors. This complicates the assignment of one single exclusion criterion to each record. The choice was therefore made to display only the number of studies for each exclusion criterion for the third screening phase (i.e. full-text screening). Regarding the number of records submitted but not yet published (n=1), this should indeed be visible in Figure 2. Unfortunately, some of the text in this Figure disappeared when transferring to a different format. This issue has been resolved.

**Additional comments**

The basis of excluding subgroups with a sample size of <20 in a given record (lines 229-230) from analysis for a given test is not entirely clear since narrative analysis was chosen over meta-analysis. Is this related to some concern for bias? Statistical consideration? A short discussion of the associated statistical methods may be of interest.

Response: Thank you for this question. The decision to exclude subgroups with a sample size <20 was made initially as a precautionary measure to ensure statistical robustness, before it became clear that a narrative analysis would be more appropriate than a meta-analysis. Although the analysis became narrative in nature, the exclusion of very small subgroups continues to safeguard the overall validity of the conclusions by reducing the risk of bias and ensuring a more reliable and consistent interpretation of the evidence.

Following adaptations were made on lines 232-234: “If, in a subgroup, the added sample size in a cyst type class was below 20, the concerning data of this class was excluded from analysis *to reduce the risk of bias and ensure a more reliable and consistent interpretation of the evidence.”*

Consistently reporting all three values in the narrative text or choosing one (e.g. accuracy, Sn or Sp) to report with guidance to consult Table 1 or S2 for the others not reported would make
the presented results clearer.

Response: Thank you for this remark. We have made the decision to focus in our Results section on the reporting of Sn and Sp, and have added extra guidance for readers to consult either Table 1, Table 2, or Supplementary Table S2.

Lines 299-300: We have clarified here that in following lines first results will be discussed that can be found in Table 1.

Lines 299-332: The order in which results are discussed was adapted to match the order in which tests are ranked in Table 1.

Lines 300-302: “The Western Blot based on LLGP ~~performed well~~ *showed sensitivities of 81.1-100.0%* in detection of parenchymal active multiple cysts *~~(81.1-100.0%)~~* [31, 32, 53]”

Lines 307-310: “The newly developed multi-antigen print immunoassay (MAPIA) based on three recombinant and synthetic antigens, ~~stands out for detection of~~ *shows notable sensitivity for* parenchymal active single and multiple cysts (100.0%) and extraparenchymal cysts in the subarachnoid space (100.0%)”

Lines 312-315: “The Western Blot based on rTsEndoB1 [60], and the Western Blot based on rTsAg5 [33], *display promising sensitivities for detection of parenchymal active (71.9%) [33] and inactive (72.5%) [60] multiple cysts and extraparenchymal cysts (96.4%) [33]*, however limited specificity in the control group (75.7%) [33], and cross-reactivities (e.g. with taeniasis, hydatidosis) could limit their use [60].”

Line 318-320: “Similarly, urine-based antigen-detecting tests (antigen ELISA and POC dipstick test formats) presented ~~accuracies~~ *sensitivities* of 96.2% and 96.7%”

Lines 333-336: “Some reports that evaluated commercial kits, … presented too little ~~accuracy~~ data”

Lines 339-340: “*Also displayed in Table 1* are five studies evaluating promising rapid tests or POC tests”

Tables 1 and 2, S2 Table: Title changed to “Test ~~accuracy~~ *sensitivity and specificity*”

Lines 471-472: “While these tests showed overall higher ~~accuracy~~ *sensitivity* for multiple cysts than antigen-detecting tests”

Lines 477-479: “The newly developed MAPIA based on *T. solium* recombinant and synthetic antigens [31], seemingly exceeded ~~accuracy~~ *sensitivity* results of other test formats for single cyst detection.”

Lines 483-485: “Some test results are promising but were excluded from Tables 1 and 2 due to inadequate characterization or insufficient sample size. *These results can be consulted in the supplementary S2 Table.*”

Line 366 is at odds with results presented in lines 272-3 (504 evaluated tests reported) and  276-7 (53 studies reported as included comprising 123 tests evaluated)

Response: Thank you for pointing out this error in the manuscript. Results presented in lines 277-279 are correct. Therefore, numbers in line 404-406 were adapted:

“Of the 169 records selected for data collection evaluating a total of *504* tests, *53* studies assessing *123* tests were ultimately included with high and/or low confidence.”

Line 403 figure reference: did this mean Fig 3 Q4 (concerning RoB analysis)?

Response: This is indeed correct (Fig *3* Q4). We are most thankful to you to point out this error.

It is less clear why some of the records previously evaluated by Cardona-Arias (e.g. Villota G, 2003; Rao V, 2011; Cho S, 1986; etc.) did not meet inclusion criteria for this study as they otherwise would appear to have met the prior review's requirements for inclusion and meta-analysis. It would be interesting supplementary material (if possible within limits for this article submission) to also see a list of the studies excluded after full-text review.

Response: Thank you, we highly appreciate your interest and suggestion. We have added a list of records excluded in the third screening phase (full-text screening), with the reason of exclusion. The list can be found in the supplementary materials, as S4 Table. Also, an overview of the selection procedure of records (confidence scaling + further selection) has been added as a supplementary file: S5 Table.

A reference to S4 Table and S5 Table is also made in the Supporting information section of the manuscript.

Line 788: *S4 Table. Third screening phase (full-text screening) excluded records with reason of exclusion.*

Line 789: *S5 Table. Confidence scaling and record selection procedure.*

I want to emphasize that the article is overall readily intelligible, though there are a few minor areas where copy editing would be “cosmetically” appealing or could enhance clarity (e.g. line 52 “fulfil” [sic] is misspelled; line 373 “synthetization” [sic] is perhaps used in lieu of "synthesis"; lines 420-421 word choice “LLGP Western Blot approximates high sensitivity and specificity aspirations” is not entirely clear in its meaning).

Response: Thank you, we appreciate your comments here. We have made following adjustments:

Line 52: “The lentil lectin-bound glycoprotein enzyme-linked immunoelectrotranfser blot seems to *fulfill* high accuracy standards…”

Lines 213-214: “…when data did not *fulfill* the criteria for abovementioned classification, the concerned entity was excluded from further analysis.”

Lines 413: “specified data is needed on reagent procurement and preparation/*synthesis* method”

Lines 466-468: “For detection of parenchymal active multiple cysts, the antibody-detecting LLGP Western Blot *approaches the high accuracy standards*, as do antigen-detecting test formats.”

Table 1 label “Par Act 1” mismatches with caption label “par act sing” for single active parenchymal lesions.

Response: We thank you for noticing this. The mismatching caption labels were adjusted in Tables 1 and 2.

Authors have not included tests on CSF in their study, while this matrix is regularly used in the diagnosis of NCC. It may be appropriate to add a short note in the discussion (or introduction) section on why this matrix was not included. Collecting CSF is more invasive than blood sampling, and therefore less practical to use in field conditions.

Response: We thank you for this suggestion, and have adapted/added the following:

Lines 121-124: “Interestingly, the use of urine as a parasite antigen source has also been explored in the development of immunological tests, providing an alternative *to* tests that depend on more invasive blood collection [17, 18]. *Tests using cerebrospinal fluid samples were not included in this review, as they require an even more invasive approach and are therefore not applicable in field conditions*.”
